# Design, analysis, and experimental research on installation robot for hanging column

**DOI:** 10.1038/s41598-026-54684-w

**Published:** 2026-06-09

**Authors:** Xiaoqiang Wang, Xijing Zhu, Xiang Li, Jingyu Yang

**Affiliations:** 1 Shanxi Key Laboratory of Semiconductor Ultraprecision Machining Technology and Intelligent Equipment, North University of China, Taiyuan, 030051 China; 2 713th Research Institute of China State Shipbuilding Corporation Limited, Zhengzhou, 450015 China; 3 CSSC Haiwei Tech Co. LTD, Zhengzhou, 450001 China

**Keywords:** Hanging column, Mechanized and automated, Robot, Kinematics analysis, Inverse kinematics solution, Joint trajectory planning, Engineering, Mathematics and computing

## Abstract

To address the challenges of labor-intensive operations, high safety risks, and low operational efficiency in the installation of hanging columns within tunnels, we propose a robotics-based mechanized and automated solution. First, integrated operational equipment is designed to perform hoisting, gripping, lifting, and installation. Next, the robot linkage coordinate system is established via the modified D–H parameter method to obtain a forward kinematic model and simulate the workspace. To improve the accuracy of solving the inverse kinematic solution, a hybrid approach combining algebraic methods and genetic algorithms is proposed, and the optimal inverse solution is selected on the basis of the shortest path. Furthermore, to avoid impact and vibration while achieving optimal efficiency, a time-optimal trajectory based on seventh-degree polynomial interpolation is presented. Finally, experiments validation was conducted. The results show that the robot’s workspace fully covers the required operational range. The proposed method achieves high accuracy, with orientation accuracy better than 10^−6^ and position accuracy better than 10 × 10^−3^. The motion of the joint is smooth and shock-free. The experiments confirm the equipment’s capability to efficiently complete installation tasks, verifying the feasibility of the proposed solution. The research results provide a theoretical foundation for equipment design and offer important references for subsequent improvements.

**Supplementary Information:**

The online version contains supplementary material available at 10.1038/s41598-026-54684-w.

## Introduction

High-speed railways (HSR) stand as a cornerstone of modern transportation. The rapid expansion of HSR network has accelerated the flow of production factors-such as capital, technology, and labor-optimized inter-city resource allocation, and significantly contributed to economic growth, labor income, employment, and industrial advancement^1,2^. By 2035, China’s HSR network is projected to reach 70,000 km. However, due to China’s complex geological structure, diverse terrain, and undulating topography characterized by numerous hills and mountains, a significant number of tunnels are required along HSR lines. For example, the Chengdu–Chongqing Central HSR features 39 tunnels with a total 81 km, the LWZH-2 section of the LiuWu HSR comprises 40 tunnels with a combined length of 61 km, and the ZhangJiHuai HSR includes 117 tunnels spanning 165 km.

**Fig. 1:**
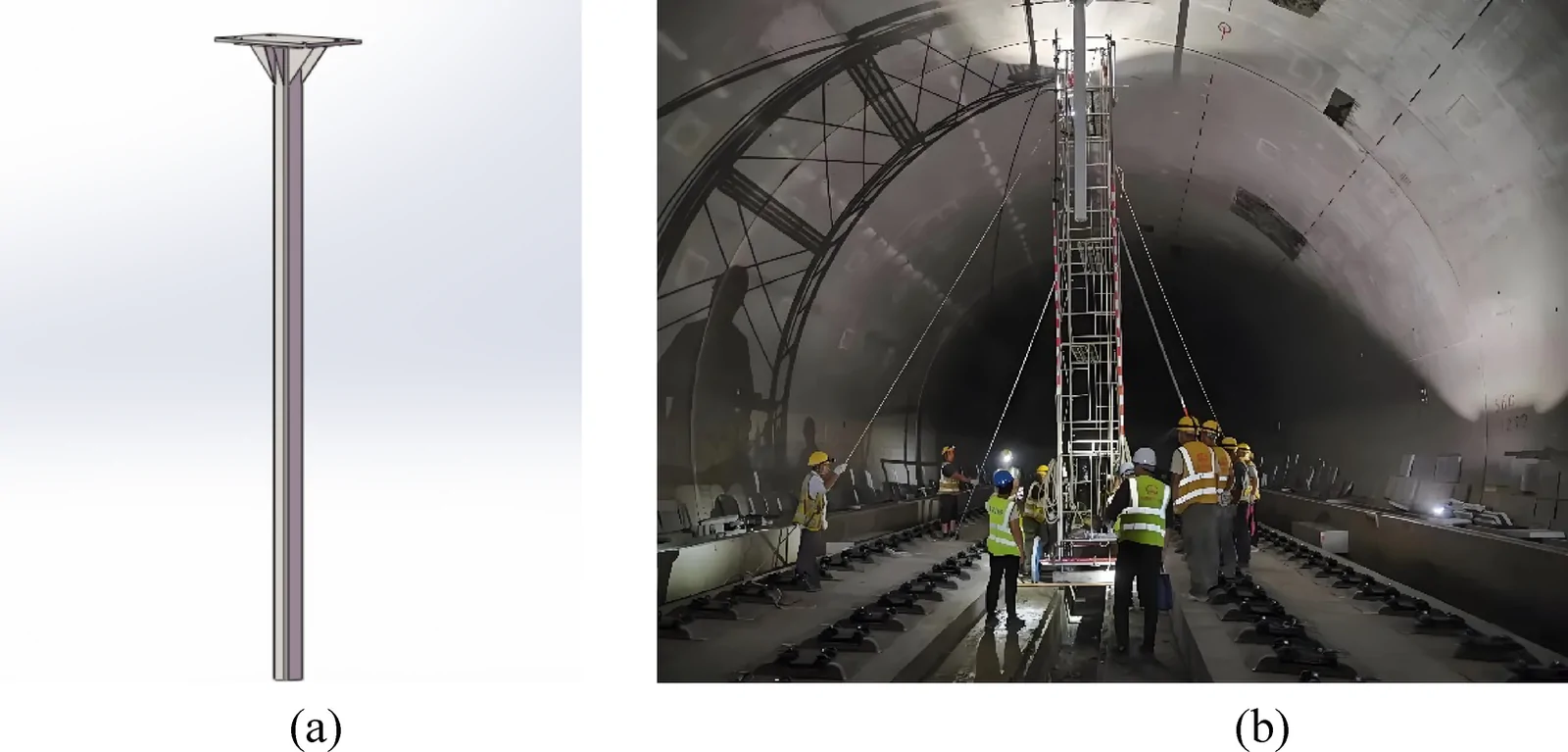
Three-dimensional (3D) model of HC and on-site installation picture. (**a**) 3D model of HC; (**b**) Traditional method for installing HC.

The hanging column (HC) is a critical component of the catenary system in tunnels. It serves to suspend and position the catenary system, bear mechanical loads, and ensure stable contact between the pantograph and the contact, thereby guaranteeing a reliable power supply for the moving railway^3^. As illustrated in Fig. 1a, the HC measures 4 m in length, has a cross-section of 120 mm × 160 mm, and weighs approximately 180 kg. It is secured to installation grooves at the tunnel crown, located approximately 9 m above the ground, using four bolts. The HC is installed at 3 m intervals along the tunnel, which means that more than 300 HC need to be installed per kilometer. However, the confined space and transportation challenges within tunnels pose significant obstacles to the progress, quality, and safety of installation projects. As shown in Fig. 1b, traditional HC installation methods rely on scaffolding and ladder trucks. Workers must manually lift HCs to the tunnel crown with the aid of lifting tools and then climb up to perform fixation. This conventional process is labor-intensive, inefficient, and hazardous, rendering it inadequate for the demands of modern construction.

Robots, as an emerging technology, can assist or replace humans in performing diverse tasks, thereby enhancing productivity, mechanization, and safety while effectively alleviating labor shortages. Robots are widely deployed in sectors such as construction and building^4–6^, agricultural planting and harvesting^7,8^, processing and manufacturing^9,10^, medical and health^11,12^, firefighting and rescue^13,14^, military missions^15,16^, and deep-sea exploration^17^. They have also been strategically introduced for HSR maintenance and operations^18,19^. To improve installation efficiency, conserve resources, and ensure construction safety, this paper proposes a novel integrated operational equipment designed to automate hoisting, gripping, transport, and assembly tasks within tunnels. This equipment aims to eliminate manual intervention, ensuring precise construction control and management. Comprising a chassis vehicle, a crane, an installation robot, an aerial work platform, and a power system, the equipment leverages automation for complex tasks. Given that the structures and movements of the auxiliary components are relatively straightforward, this study focuses primarily on the research and analysis of the installation robot.

Inverse kinematic analysis is fundamental to robot trajectory planning, involving the determination of joint variables from a given pose matrix. Common solution methods include algebraic^20^, geometric^21^, iterative^22^, and intelligent algorithms^23^. While algebraic methods can be complex and cumbersome, geometric methods are often limited by structural constraints, and iterative methods may encounter singularity issues. In contrast, intelligent algorithms offer rapid solution speeds and strong versatility. The genetic algorithm (GA), a widely used population-based intelligent method, simulates biological evolution processes such as natural selection, reproduction, crossover, and mutation. Starting from an initial population, GA iteratively evolves individuals to better fit the environment, eventually stabilizing to find an optimal or near-optimal solution^24,25^. However, standard GA often suffers from limited computational accuracy and a tendency to converge prematurely into local optima. To overcome these drawbacks, this paper proposes a hybrid method combining algebraic and genetic algorithms (AGA) to significantly enhance computational accuracy.

To ensure precise robot operation and mitigate issues arising from kinematic singularities and redundancy, joint trajectories are planned within the joint space. Common trajectory planning techniques include cubic polynomial interpolation^26^, fifth-order polynomial interpolation^27^, seventh‐order polynomial function^28^, B-splines^29,30^, and multi-segment interpolation^31,32^. Herein, we introduce a time-optimal trajectory planning method based on seventh-degree polynomial interpolation, subject to constraints on joint velocity, acceleration, and jerk. This approach ensures a smooth and continuous trajectory, minimizes vibration and impact, and achieves time optimality.

The rest of this paper is organized as follows: “Installation robot design” section briefly introduce the main components and workflow of integrated operational equipment and installation robot. “Kinematic analysis and trajectory planning” section details the kinematic analysis and trajectory planning for the installation robot. “Experiment and discussion” section focuses on the experimental validation. “Conclusion” section summarizes the key findings and conclusions.

## Installation robot design

### Mechanical structure design

**Fig. 2:**
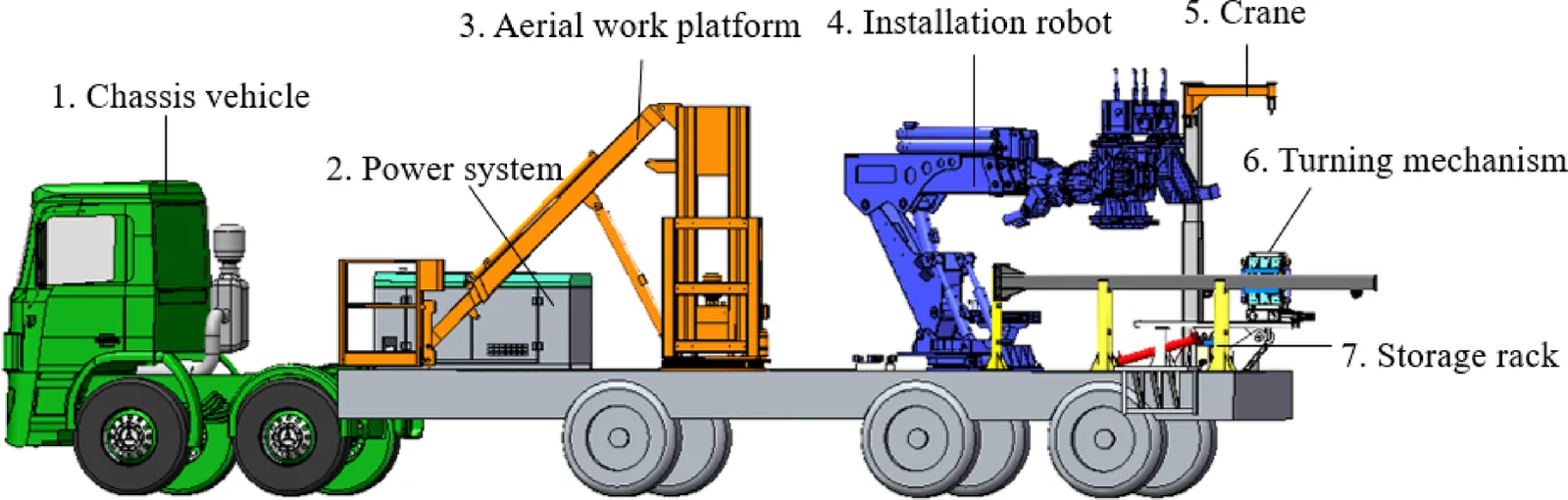
The 3D model of the integrated installation equipment.

Based on the specific operational environment and functional requirements, an integrated operation equipment dominated by the installation robot was designed. As illustrated in Fig. 2, the equipment primarily comprises chassis vehicle, power system, aerial work platform, installation robot, crane, turning mechanism, and storage rack. To facilitate transportation, the overall height of the equipment in its retracted state is limited to 3.2 m.

The chassis vehicle serves as the foundational support for all components, enabling both long-distance transit and short-range maneuvering during operations. It features a rated load capacity of 10 t, with a carrying platform measuring 10 m in length and 2.55 m in width, and a ground clearance of 1.55 m. The power system acts as the primary energy and hydraulic source for the entire equipment suite, consisting mainly of an engine, starter battery, fuel tank, gear pump, and hydraulic reservoir. The aerial work platform, with a rated load capacity of 300 kg and an adjustable working height ranging from 0.9 to 6.8 m, is designed to transport two workers to the tunnel crown. The installation robot is responsible for grasping HCs, transporting them to the installation position, and assisting in the fixation process. The crane, capable of lifting 300 kg, transfers HCs from the ground to an intermediate height of up to 3 m. The storage rack provides temporary storage for HCs, while the turning mechanism flips the HCs from the storage orientation to the robot’s gripping position. Given its critical role, this paper focuses specifically on the research and analysis of the installation robot.

**Fig. 3:**
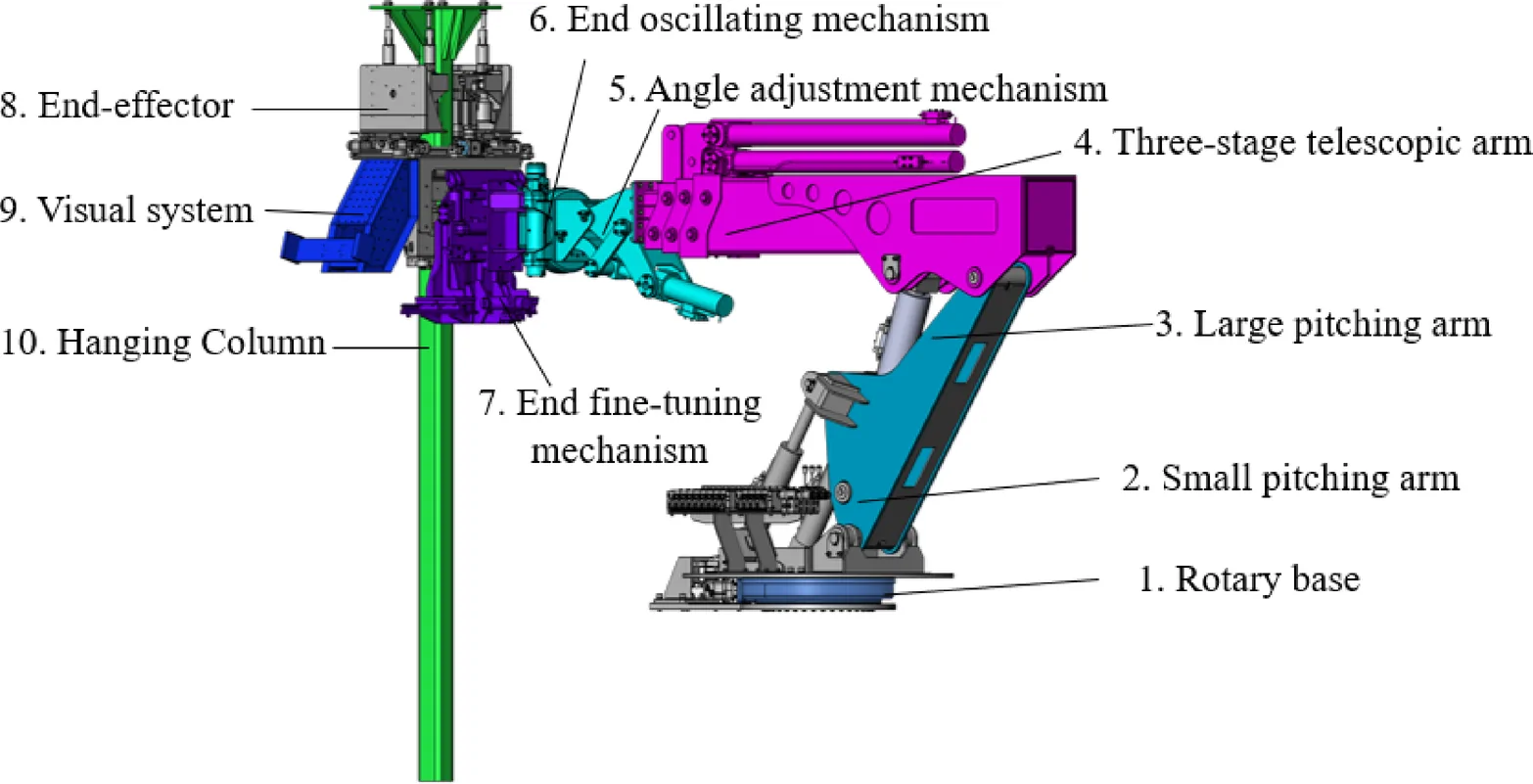
3D model of installation robot.

The installation robot, mounted on the chassis, comprises a rotary base, a small pitch arm, a large pitch arm, a three-stage telescopic arm, an angle adjustment mechanism, an end oscillating mechanism, an end fine-tuning mechanism, an end-effector, and a visual system. The robotic arm facilitates the spatial movement and pose adjustment of the end-effector from the grasping zone to the installation point. The end-effector is responsible for gripping the HC, aligning its position, and tightening the fixing bolts. The visual system provides real-time detection of the HC’s location. The 3D model of installation robot is presented in Fig. 3.

**Fig. 4:**
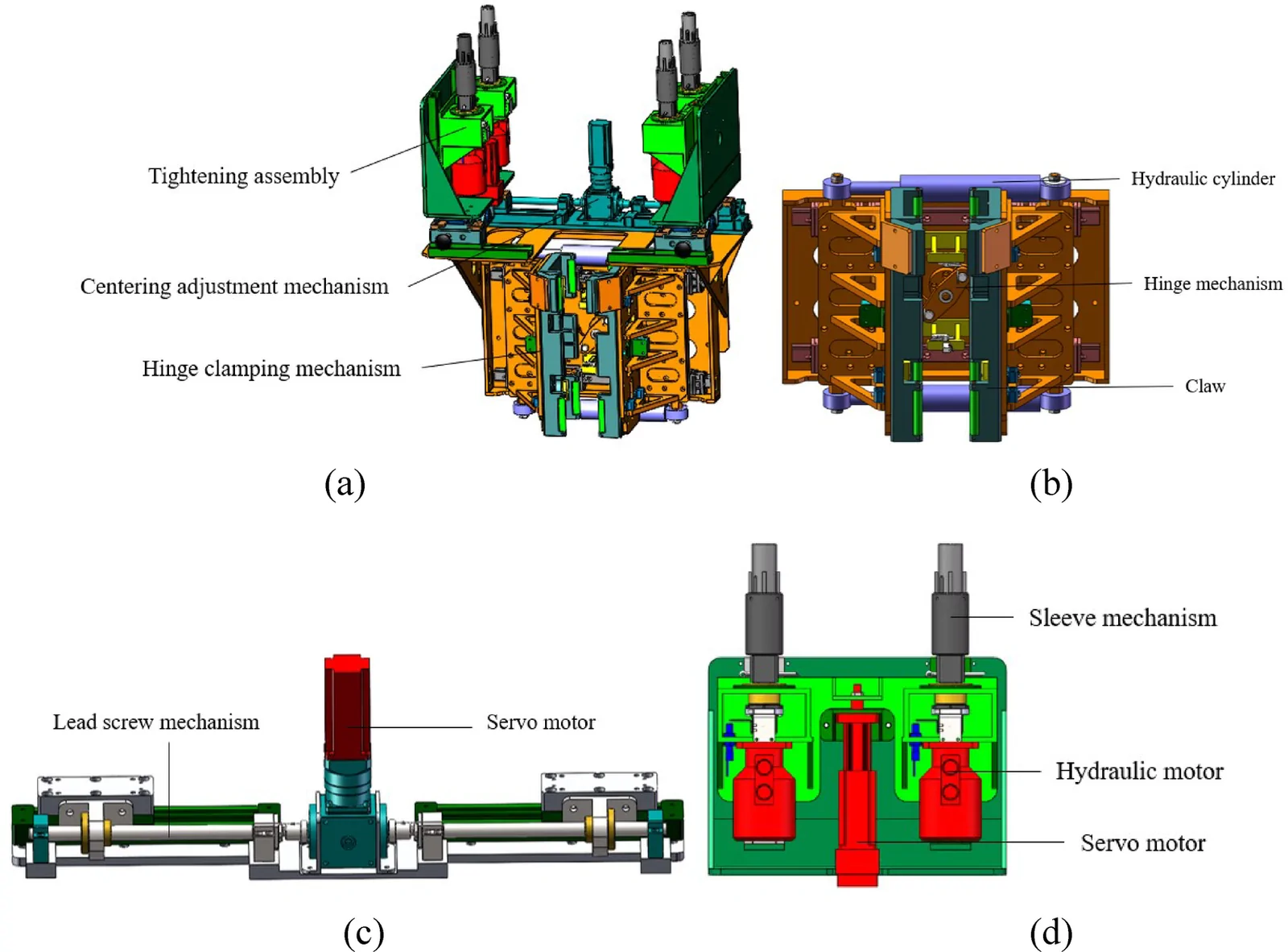
3D model of the end-effector. (**a**) The end-effector; (**b**) The hinge clamping mechanism; (**c**) The alignment adjustment mechanism; (**d**) The tightening assembly.

The end-effector primarily consists of a hinge clamping mechanism, an alignment adjustment mechanism, and a tightening assembly. Its overall structural 3D model is shown in Fig. 4a. The hinge clamping mechanism, designed to grasp the HC, mainly comprises a hinge mechanism, claws, and hydraulic cylinders, as illustrated in Fig. 4b. During operation, the hydraulic cylinders drive the hinge mechanism, which in turn actuates the claws to secure the HC. The alignment adjustment mechanism, designed to positions the tightening assembly so that the sleeve center coincides with the bolt hole center, features a lead screw driven by a servo motor, as shown in Fig. 4c. Upon activated, the motor rotates the screw, translating the tightening assembly along the axis to achieve precise alignment. The tightening assembly, designed to tighten and secure bolts, primarily consists of a servo motor, a hydraulic motor, and a sleeve mechanism, as shown in Fig. 4d. In operation, the servo motor translates the sleeve vertically to engage the bolt, while the hydraulic motor simultaneously or subsequently rotates the sleeve to tighten and fix the bolt.

### Operation process

**Fig. 5:**
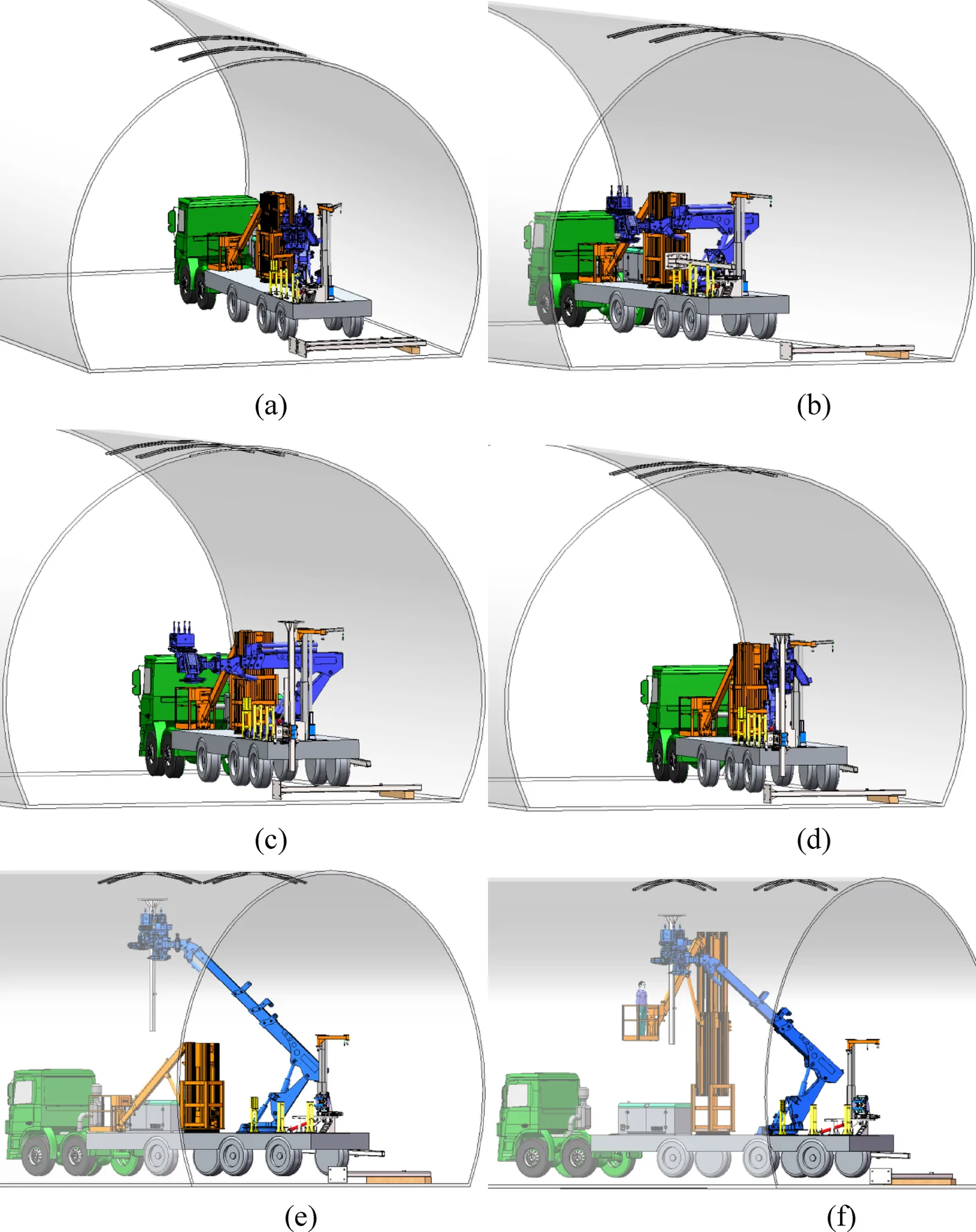
The mechanized and automated workflow for HC installation. (**a**) Parking; (**b**) Hoisting and storage; (**c**) Rotating; (**d**) Griping; (**e**) Lifting; (**f**) Installing.

The mechanized workflow of HC installation is illustrated in Fig. 5. First, the chassis vehicle maneuvers into the work area and positions itself. Second, the crane hoists an HC from the ground to a height of approximately 2.5 m and places it onto the storage rack. Third, the turning mechanism grasps the HC and rotates it by 90°. Fourth, the installation robot grips the HC. Fifth, the installation robot lifts HC to the tunnel crown and moves it to the precise installation position guided by the vision system. Finally, workers are elevated by the aerial work platform to the vicinity of the installation point, where they complete the fixation of the HC in coordination with the robot.

## Kinematic analysis and trajectory planning

### Forward kinematics analysis and modelling

Based on the 3D model, the installation robot is simplified into a link-joint kinematic chain with sequential numbering. Specifically: the rotary base is simplified into joint J1 and link L1, small pitching arm is simplified into joint J2 and link L2, large pitching arm is simplified into joint J3 and link L3, three-stage telescopic arm is simplified into joint J4 and link L4, angle adjustment mechanism is simplified into joint j5 and link L5, end fine-tuning mechanism is simplified into joint J6 and link L6. Joints J1, J2, J3, J5, J6 are revolute joints, while joint J4 is a prismatic joint.

**Fig. 6:**
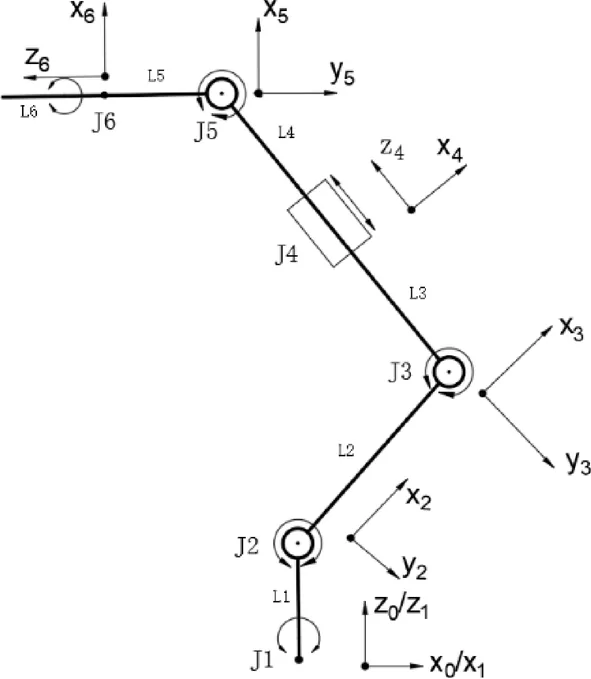
The simplified diagram and coordinate systems of installation robot.

The modified D–H parameter convention is employed to establish coordinate frames at the proximal end of each link. The robot base is fixed and stationary, and the coordinate frame {0} rigidly attached to the base as the reference frame. The coordinate axes are defined as follows: the Z-axis is aligned with the axis of rotation for revolute joints or the direction of motion for prismatic joints, the X-axis is defined as the common normal perpendicular to the adjacent Z-axes, the Y-axis is determined using the right-hand rule. The simplified kinematic diagram and coordinate systems are illustrated in Fig. 6.

The D–H parameters of installation robot consist of four parameters: link angle α_i−1_, link length a_i−1_, joint angle θ_i_, and joint distance di. These parameters, along with the joint variation ranges, are listed in Table 1.

**Table 1:** D–H parameter table.

Link	θ_i_	d_i_	a_i−1_	α_i−1_	Variation rang
1	θ_1_	0	0	0	(− 70°, 70°)
2	θ_2_	0	0	− 90°	(− 70°, − 30°)
3	θ_3_	0	1630	0	(− 10°, 30°)
4	0	d_4_	0	90°	(2900, 8100)
5	θ_5_	0	0	− 90°	(− 70°, 0°)
6	θ_6_	1340	0	90°	(− 90°, 90°)

The transformation from frame {i − 1} to {i} involves the following sequential operations: rotation by α_i−1_ about the x_i−1_ axis, translation by a_i−1_ along the x_i−1_ axis, rotation by θ_i_ about the z_i_ axis, and translation by di along the z_i_ axis. Following the principle of post-multiplication, the homogeneous transformation matrix representing the continuous transformation from {i − 1} to {i} is expressed as

1 $$\begin{aligned} {}_{i}^{i - 1} T & = Rot\left( {x,{ }\alpha_{i - 1} } \right)Trans\left( {a_{i - 1} ,0,0} \right)Rot\left( {z,{ }\theta_{i} } \right)Trans\left( {0,0,d_{i} } \right) \\ & = \left[ {\begin{array}{*{20}c} {c{\uptheta}_{i} } & { - s{\uptheta}_{i} } & 0 & {{\mathrm{a}}_{{{\mathrm{i}} - 1}} } \\ {s{\uptheta}_{i} c\alpha_{i - 1} } & {c{\uptheta}_{i} c\alpha_{i - 1} } & { - s\alpha_{i - 1} } & { - d_{i} s\alpha_{i - 1} } \\ {s{\uptheta}_{i} s\alpha_{i - 1} } & {c{\uptheta}_{i} s\alpha_{i - 1} } & {c\alpha_{i - 1} } & {d_{i} c\alpha_{i - 1} } \\ 0 & 0 & 0 & 1 \\ \end{array} } \right]. \\ \end{aligned}$$

By substituting the D–H parameters of robot listed in Table 1 into Eq. (1), the transformation matrices between adjacent links are calculated as

2 $$\left\{ {\begin{array}{*{20}l} {{}_{1}^{0} T = \left[ {\begin{array}{*{20}c} {c\uptheta _{1} } & { - s\uptheta _{1} } & 0 & 0 \\ {s\uptheta _{1} } & {c\uptheta _{1} } & 0 & 0 \\ 0 & 0 & 1 & 0 \\ 0 & 0 & 0 & 1 \\ \end{array} } \right] } \hfill & {{}_{2}^{1} T = \left[ {\begin{array}{*{20}c} {c\uptheta _{2} } & { - s\uptheta _{2} } & 0 & 0 \\ 0 & 0 & 1 & 0 \\ { - s\uptheta _{2} } & { - c\uptheta _{2} } & 0 & 0 \\ 0 & 0 & 0 & 1 \\ \end{array} } \right]} \hfill \\ {{}_{3}^{2} T = \left[ {\begin{array}{*{20}c} {c\uptheta _{3} } & { - s\uptheta _{3} } & 0 & {1630} \\ {s\uptheta _{3} } & {c\uptheta _{3} } & 0 & 0 \\ 0 & 0 & 1 & 0 \\ 0 & 0 & 0 & 1 \\ \end{array} } \right]} \hfill & {{}_{4}^{3} T = \left[ {\begin{array}{*{20}c} 1 & 0 & 0 & 0 \\ 0 & 0 & { - 1} & { - d_{4} } \\ 0 & 1 & 0 & 0 \\ 0 & 0 & 0 & 1 \\ \end{array} } \right]} \hfill \\ {{}_{5}^{4} T = \left[ {\begin{array}{*{20}c} {c\uptheta _{5} } & { - s\uptheta _{5} } & 0 & 0 \\ 0 & 0 & 1 & 0 \\ { - s\uptheta _{5} } & { - c\uptheta _{5} } & 0 & 0 \\ 0 & 0 & 0 & 1 \\ \end{array} } \right]} \hfill & {{}_{6}^{5} T = \left[ {\begin{array}{*{20}c} {c\uptheta _{6} } & { - s\uptheta _{6} } & 0 & 0 \\ 0 & 0 & { - 1} & { - 1340} \\ {s\uptheta _{6} } & {c\uptheta _{6} } & 0 & 0 \\ 0 & 0 & 0 & 1 \\ \end{array} } \right]} \hfill \\ \end{array} } \right..$$

According to the principle of post-multiplication, sequentially multiplying these adjacent transformation matrices yields the homogeneous transformation matrix of the end-effector relative to the base frame, as expressed in Eq. (3).

3 $${}_{6}^{{{ }0}} T = { }{}_{1}^{0} T{ }{}_{2}^{1} T{ }{}_{3}^{2} T{ }{}_{4}^{3} T{ }{}_{5}^{{{ }4}} T{}_{6}^{{{ }5}} T{ } = \left[ {\begin{array}{*{20}c} {{\mathrm{n}}_{x} } & {{\mathrm{o}}_{x} } & {{\mathrm{a}}_{x} } & {{\mathrm{p}}_{x} } \\ {{\mathrm{n}}_{y} } & {{\mathrm{o}}_{y} } & {{\mathrm{a}}_{y} } & {{\mathrm{p}}_{y} } \\ {{\mathrm{n}}_{z} } & {{\mathrm{o}}_{z} } & {{\mathrm{a}}_{z} } & {{\mathrm{p}}_{z} } \\ 0 & 0 & 0 & 1 \\ \end{array} } \right]$$

$$\left\{ {\begin{array}{*{20}l} {{\mathrm{n}}_{{\mathrm{x}}} = c\uptheta _{1} c\uptheta _{6} {\mathrm{c}}\left( {\uptheta _{2} +\uptheta _{3} +\uptheta _{5} } \right) - s\uptheta _{1} s\uptheta _{6} } \hfill \\ {{\mathrm{o}}_{{\mathrm{x}}} = - c\uptheta _{1} s\uptheta _{6} {\mathrm{c}}\left( {\uptheta _{2} +\uptheta _{3} +\uptheta _{5} } \right) - s\uptheta _{1} c\uptheta _{6} } \hfill \\ {{\mathrm{a}}_{{\mathrm{x}}} = c\uptheta _{1} {\mathrm{s}}\left( {\uptheta _{2} +\uptheta _{3} +\uptheta _{5} } \right)} \hfill \\ {{\mathrm{p}}_{{\mathrm{x}}} = c\uptheta _{1} \left[ {1340 \times {\mathrm{s}}\left( {\uptheta _{2} +\uptheta _{3} +\uptheta _{5} } \right) + 1630 \times {\mathrm{c}}\uptheta _{2} + {\mathrm{d}}_{4} {\mathrm{s}}\left( {\uptheta _{2} +\uptheta _{3} } \right)} \right]} \hfill \\ {{\mathrm{n}}_{{\mathrm{y}}} = s\uptheta _{1} c\uptheta _{6} {\mathrm{c}}\left( {\uptheta _{2} +\uptheta _{3} +\uptheta _{5} } \right) + c\uptheta _{1} s\uptheta _{6} } \hfill \\ {{\mathrm{o}}_{{\mathrm{y}}} = - s\uptheta _{1} s\uptheta _{6} {\text{ c}}\left( {\uptheta _{2} +\uptheta _{3} +\uptheta _{5} } \right) + c\uptheta _{1} c\uptheta _{6} } \hfill \\ {{\mathrm{a}}_{{\mathrm{y}}} = s\uptheta _{1} {\mathrm{s}}\left( {\uptheta _{2} +\uptheta _{3} +\uptheta _{5} } \right)} \hfill \\ {{\mathrm{p}}_{{\mathrm{y}}} = s\uptheta _{1} \left[ {1340 \times {\mathrm{s}}\left( {\uptheta _{2} +\uptheta _{3} +\uptheta _{5} } \right) + 1630 \times {\mathrm{c}}\uptheta _{2} + {\mathrm{d}}_{4} {\mathrm{s}}\left( {\uptheta _{2} +\uptheta _{3} } \right)} \right]} \hfill \\ {{\mathrm{n}}_{{\mathrm{z}}} = - c\uptheta _{6} {\mathrm{s}}\left( {\uptheta _{2} +\uptheta _{3} +\uptheta _{5} } \right)} \hfill \\ {{\mathrm{o}}_{{\mathrm{z}}} = s{\uptheta}_{6} {\mathrm{s}}\left( {\uptheta _{2} +\uptheta _{3} +\uptheta _{5} } \right)} \hfill \\ {{\mathrm{a}}_{{\mathrm{z}}} = {\mathrm{c}}\left( {\uptheta _{2} +\uptheta _{3} +\uptheta _{5} } \right)} \hfill \\ {{\mathrm{p}}_{{\mathrm{z}}} = 1340 \times {\mathrm{c}}\left( {\uptheta _{2} +\uptheta _{3} +\uptheta _{5} } \right) - 1630 \times {\mathrm{s}}\uptheta _{2} + {\mathrm{d}}_{4} {\mathrm{c}}\left( {\uptheta _{2} +\uptheta _{3} } \right)} \hfill \\ \end{array} } \right.$$

On the basis of the D–H parameters in Table 1, a forward kinematics simulation model was established using MATLAB’s Robotics Toolbox, as shown in Fig. S1. To validate the analytical model, three sets of joint variables were randomly selected and substituted into both Eq. (3) and the simulation model to solve for the end pose matrix. The comparative results are presented in Table S1. The data show that the solutions from both methods are identical, confirming the validity of the derived kinematic model.

### Workspace analysis

Based on operational requirements, the robot’s working range is defined as follows: the robot should reach a radius of 2.8–3.5 m and a height of 2.8–3.5 m at the grasping position, and a radius of 1.8–4.2 m and a height of 4.3–8 m at the installation position. These requirements are visualized in MATLAB and presented in Fig. S2.

To verify whether the designed installation robot meets these specifications, the reachable workspace was analyzed using the Monte Carlo method^33^. This involves randomly sampling joint variables within their respective motion limits and substituting them into the kinematic equations to determine the set of all reachable positions. The procedure is as follows: First, the simulation model was established (Fig. S1). Second, random numbers r_1_, r_2_…r_n_ were generated in the interval [0, 1] to calculate corresponding joint variables within their limits. Taking joint J1 as an example, if a random value r_i_ is selected, the joint variables is calculated as q = θ_1min_ + r_i_ × (θ_1max_ − θ_1min_). Third, these n sets of random joint variables ware substituted into the forward kinematics equations to obtain n sets of spatial coordinates. Finally, these coordinates were plotted to generate a point cloud representing the robot’s workspace.

**Fig. 7:**
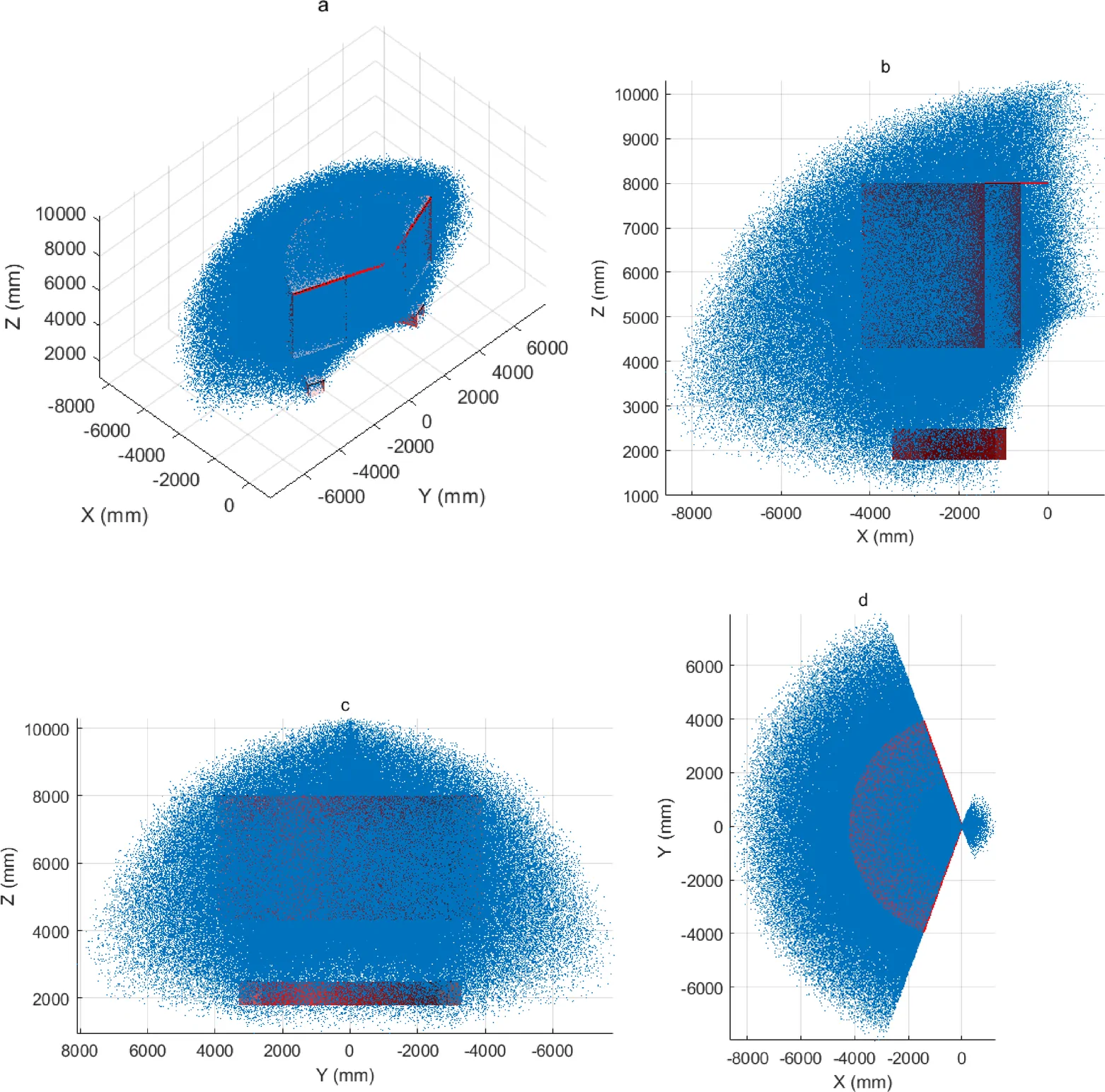
Workspace of installation robot. (**a**) Global Workspace; (**b**) X0Z Plane; (**c**) YOZ Plane; (**d**) X0Y Plane.

With *n* = 300,000, the workspace was simulated and overlaid with the required working range, and the results are shown in Fig. 7. The simulated workspace boundaries are: − 8627 mm ≤ X ≤ 1342 mm, − 8153 mm ≤ Y ≤ 8095 mm, 949 mm ≤ Z ≤ 10,312 mm. The workspace (blue points) fully encompasses the required working range (red area), confirming that the design meets the operational requirements. Moreover, the generated workspace significantly exceeds the requirements, suggesting that the stroke of the prismatic joint J4 can be optimized. By iteratively adjusting the travel range of J4 and re-simulating, the optimal range was determined to be (2900, 7600). This optimization reduces the joint stroke by 6.17% while still satisfying all working range requirements. The optimized simulation results are presented in Fig. S3, with the new workspace boundaries: − 8143 mm ≤ X ≤ 1266 mm, − 7694 mm ≤ Y ≤ 7643 mm, 987 mm ≤ Z ≤ 9839 mm.

### Solving the inverse kinematics

#### Algorithm design and analysis

**Fig. 8:**
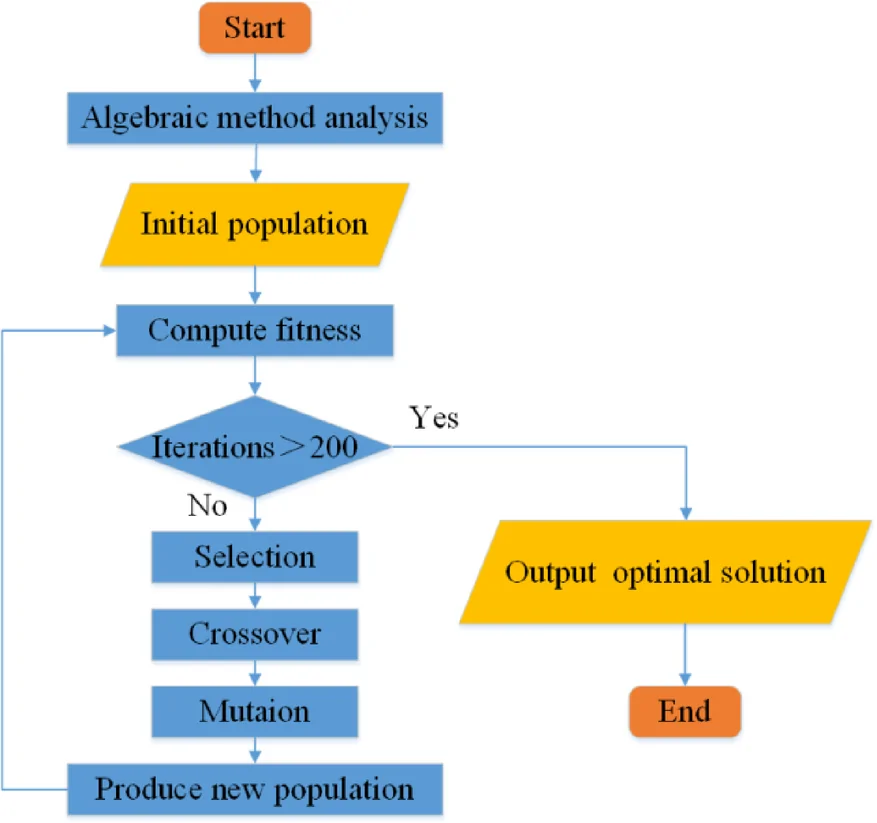
Flowchart of the AGA.

Inverse kinematics analysis involves determining the joint variables corresponding to a given end pose matrix. In this paper, the AGA method is proposed to solve the robot’s inverse kinematics problem, and the algorithm’s flowchart is illustrated in Fig. 8.

- Algebraic method analysis

An algebraic analysis is first performed to directly solve for specific joint variables or to identify coupling constraints among joints based on the kinematic equations and the pose matrix. This step effectively reduces the number of unknowns and narrows the solution search space.

- Initial population

The joint variation ranges for the installation robot are listed in Table 1. The initial population size is N. Each individual in the population consists of six joint variables, which are randomly initialized within their respective limits using MATLAB’s “randi function” as follows:

i = 1, 2, 3, …, N

Population (i, 1) = randi (− 70°, 70°).

Population (i, 2) = randi (− 70°, − 30°).

Population (i, 3) = randi (− 10°, 30°).

Population (i, 4) = randi (2900 , 7600).

Population (i, 5) = randi (− 70° , 0).

Population (i, 6) = randi (− 90° , 90°).

- Fitness function

The current joint variables of the i-th individual are substituted into the forward kinematics Eq. (3) to obtain the actual pose matrix as T_i_. Given the target pose matrix T, the error between T and T_i_ is calculated as Eq. (4).

4 $${\mathrm{T}} - {\mathrm{T}}_{{\mathrm{i}}} = \left[ {\begin{array}{*{20}c} {\Delta {\mathrm{n}}_{x} } & {\Delta {\mathrm{o}}_{x} } & {\Delta {\mathrm{a}}_{x} } & {\Delta {\mathrm{p}}_{x} } \\ {\Delta {\mathrm{n}}_{y} } & {\Delta {\mathrm{o}}_{y} } & {\Delta {\mathrm{a}}_{y} } & {\Delta {\mathrm{p}}_{y} } \\ {\Delta {\mathrm{n}}_{z} } & {\Delta {\mathrm{o}}_{z} } & {\Delta {\mathrm{a}}_{z} } & {\Delta {\mathrm{p}}_{z} } \\ 0 & 0 & 0 & 1 \\ \end{array} } \right] = \left[ {\begin{array}{*{20}c} {\Delta R_{3 \times 3} } & {\Delta p_{3 \times 1} } \\ 0 & 1 \\ \end{array} } \right]$$

The fitness function is designed as Eq. (5).

5 $$\begin{aligned} {\mathrm{Fitness}}\left( {\mathrm{i}} \right) & = \beta \Delta R_{3 \times 3} + \left( {1 - \beta } \right)\Delta p_{3 \times 1} \\ & = \beta \left( {{ }\left| {\Delta {\mathrm{n}}_{x} } \right| + \left| {\Delta {\mathrm{o}}_{x} } \right| + \left| {\Delta {\mathrm{a}}_{x} } \right| + \left| {\Delta {\mathrm{n}}_{y} } \right| + \left| {\Delta {\mathrm{o}}_{y} } \right| + \left| {\Delta {\mathrm{a}}_{y} } \right| + \left| {\Delta {\mathrm{n}}_{z} } \right| + \left| {\Delta {\mathrm{o}}_{z} } \right| + \left| {\Delta {\mathrm{a}}_{z} } \right|{ }} \right) \\ & \quad + \left( {1 - \beta } \right){ }\left( {{ }\left| {\Delta {\mathrm{p}}_{x} } \right| + \left| {\Delta {\mathrm{p}}_{y} } \right| + \left| {\Delta {\mathrm{p}}_{z} } \right|{ }} \right) \\ \end{aligned}$$

where $\Delta R_{3 \times 3}$ and $\Delta p_{3 \times 1}$ represent the orientation and position error, respectively, β and 1 − β are the corresponding weighting factors.

- Selection

The fitness value of the i-th individual is then computed as Eq. (6).

6 $${\mathrm{Fitness}}\left( {\mathrm{i}} \right) = 1/\left( {{\mathrm{Fitness}}\left( {\mathrm{i}} \right) + 0.000001} \right)$$

Subsequently, the Roulette Wheel Selection method is employed. This fitness-proportionate selection technique simulates a roulette wheel to determine which individuals are selected for the next generation. Its core principle is that the selection probability of an individual is directly proportional to its fitness value. Thus, individuals with higher fitness have a greater chance of being chosen. The selection probability for the i-th individual is defined by Eq. (7).

7 $$p_{i} = \frac{{{\mathrm{Fitness}}\left( {\mathrm{i}} \right)}}{{\mathop \sum \nolimits_{j = 1}^{N} {\mathrm{Fitness}}\left( {\mathrm{j}} \right)}}$$

- Crossover

Single-point crossover is used to generate new offspring. Whether crossover occurs between two adjacent individuals i and i + 1 is determined by the crossover rate. If crossover is triggered, a crossover point is randomly selected, and the gene segments following the point are exchanged between the two individuals to create two new offspring; otherwise, the individual remains unchanged.

- Mutation

Following crossover, each individual in the population is subjected to a mutation operation based on the mutation rate. If mutation occurs, specific joint variables are randomly regenerated within their respective motion limits; otherwise, the individual remains unchanged.

#### Algorithm simulation

When the HC is transported to the installation position near the tunnel ceiling, the target end pose matrix of the robot is denoted as T.

$${\mathrm{T}} = \left[ {\begin{array}{*{20}c} {0.433013} & {0.500000} & { - 0.750000} & { - 3113.77} \\ { - 0.250000} & {0.866025} & {0.433013} & {1797.74} \\ {0.866025} & 0 & {0.500000} & {7110.79} \\ 0 & 0 & 0 & 1 \\ \end{array} } \right]$$

Through algebraic analysis, by equating the elements of the Eq. 3) with the target matrix T, the relationships shown in Eq. (8) are derived:

8 $$\left\{ {\begin{array}{*{20}l} {{\mathrm{a}}_{{\mathrm{x}}} = c\uptheta _{1} s\left( {\uptheta _{2} +\uptheta _{3} +\uptheta _{5} } \right) = - 0.750000 } \hfill \\ {{\mathrm{a}}_{{\mathrm{y}}} = s\uptheta _{1} s\left( {\uptheta _{2} +\uptheta _{3} +\uptheta _{5} } \right) = 0.433013} \hfill \\ {{\mathrm{n}}_{{\mathrm{z}}} = - c\uptheta _{6} s\left( {\uptheta _{2} +\uptheta _{3} +\uptheta _{5} } \right) = 0.866025} \hfill \\ {{\mathrm{o}}_{{\mathrm{z}}} = s\uptheta _{6} s\left( {\uptheta _{2} +\uptheta _{3} +\uptheta _{5} } \right) = 0} \hfill \\ {{\mathrm{a}}_{{\mathrm{z}}} = c\left( {\uptheta _{2} +\uptheta _{3} +\uptheta _{5} } \right) = 0.500000} \hfill \\ \end{array} } \right..$$

Within the allowable joint ranges, the following analytical solutions can be obtained:

$$\uptheta _{1} = - 30^{ \circ } ,\uptheta _{6} = 0,\uptheta _{2} +\uptheta _{3} +\uptheta _{5} = - 60^{ \circ } .$$

By integrating these analytical solutions into the "initial population " and “mutation” stages of the algorithm, the number of unknown variables is reduced from six to four. Furthermore, the search spaces for θ₂, θ₃, and θ₅ are effectively constrained, thereby improving the convergence efficiency.

The proposed AGA method was employed to solve the inverse kinematics problem. The simulation parameters were configured as follows: the number of iterations is 200, the population size *N* = 20,000, the weight of the orientation error β is 0.3, the crossover rate is 0.9, and the mutation rate is 0.03. The algorithm was executed to obtain the optimal joint configurations.

To verify repeatability, the AGA was run four times, yielding four sets of joint variables as listed in Table 2. To assess solution quality, these variables were substituted into the forward kinematic Eq. (3) to compute the actual end pose matrix Ti, and the error relative to the target pose matrix T is calculated, as summarized in Table 2. For comparison, the traditional GA was also employed to solve for the joint variables, with results presented in Table 3. In the pose matrix, the upper-left 3 × 3 block denotes orientation, and the first three elements of the fourth row denote position.

**Table 2:** The running results of the AGA.

Joint variables	End pose matrix	Errors
(− 30°, − 47°, 13°, 6335, − 26°, 0)	$$\left[ {\begin{array}{*{20}c} {0.433013} & {0.500000} & { - 0.750000} & { - 3110.16} \\ { - 0.250000} & {0.866025} & {0.433013} & {1795.65} \\ {0.866025} & 0 & {0.500000} & { 7114.06} \\ 0 & 0 & 0 & 1 \\ \end{array} } \right]$$	$$\left[ {\begin{array}{*{20}c} { - 6.88 \times 10^{ - 7} } & { - 1.11 \times 10^{ - 16} } & {1.48 \times 10^{ - 16} } & { - 1.16 \times 10^{ - 3} } \\ { - 3.33 \times 10^{ - 16} } & {4.66 \times 10^{ - 7} } & { - 6.88 \times 10^{ - 7} } & { - 1.16 \times 10^{ - 3} } \\ {4.66 \times 10^{ - 7} } & 0 & { - 2.22 \times 10^{ - 16} } & {4.60 \times 10^{ - 4} } \\ \end{array} } \right]$$
(− 30°, − 59°, 26°, 6014, − 27°, 0)	$$\left[ {\begin{array}{*{20}c} {0.433013} & {0.500000} & { - 0.750000} & { - 3114.59} \\ { - 0.250000} & {0.866025} & {0.433013} & {1798.21} \\ {0.866025} & 0 & {0.500000} & {7110.95} \\ 0 & 0 & 0 & 1 \\ \end{array} } \right]$$	$$\left[ {\begin{array}{*{20}c} { - 6.88 \times 10^{ - 7} } & { - 1.11 \times 10^{ - 16} } & {1.48 \times 10^{ - 16} } & {2.64 \times 10^{ - 4} } \\ { - 3.33 \times 10^{ - 16} } & {4.66 \times 10^{ - 7} } & { - 6.88 \times 10^{ - 7} } & {2.62 \times 10^{ - 4} } \\ {4.66 \times 10^{ - 7} } & 0 & { - 2.22 \times 10^{ - 16} } & {2.21 \times 10^{ - 5} } \\ \end{array} } \right]$$
(− 30°, − 48°, 14°, 6307, − 26°, 0)	$$\left[ {\begin{array}{*{20}c} {0.433013} & {0.500000} & { - 0.750000} & { - 3114.76} \\ { - 0.250000} & {0.866025} & {0.433013} & {1798.31} \\ {0.866025} & 0 & {0.500000} & {7110.07} \\ 0 & 0 & 0 & 1 \\ \end{array} } \right]$$	$$\left[ {\begin{array}{*{20}c} { - 6.88 \times 10^{ - 7} } & { - 1.11 \times 10^{ - 16} } & {1.48 \times 10^{ - 16} } & {3.20 \times 10^{ - 4} } \\ { - 3.33 \times 10^{ - 16} } & {4.66 \times 10^{ - 7} } & { - 6.88 \times 10^{ - 7} } & {3.17 \times 10^{ - 4} } \\ {4.66 \times 10^{ - 7} } & 0 & { - 2.22 \times 10^{ - 16} } & { - 1.02 \times 10^{ - 4} } \\ \end{array} } \right]$$
(− 30°, − 49°, 15°, 6280, − 26°, 0°)	$$\left[ {\begin{array}{*{20}c} {0.433013} & {0.500000} & { - 0.750000} & { - 3120.14} \\ { - 0.250000} & {0.866025} & {0.433013} & {1801.41} \\ {0.866025} & 0 & {0.500000} & {7106.53} \\ 0 & 0 & 0 & 1 \\ \end{array} } \right]$$	$$\left[ {\begin{array}{*{20}c} { - 6.88 \times 10^{ - 7} } & { - 1.11 \times 10^{ - 16} } & 0 & {2.05 \times 10^{ - 3} } \\ 0 & {4.66 \times 10^{ - 7} } & { - 6.88 \times 10^{ - 7} } & {2.04 \times 10^{ - 3} } \\ {4.66 \times 10^{ - 7} } & 0 & {2.22 \times 10^{ - 16} } & { - 5.99 \times 10^{ - 4} } \\ \end{array} } \right]$$

**Table 3:** The running results of the GA.

Joint variables	End pose matrix	Errors
(− 30°, − 42°, 7°, 6437, − 21°, 0)	$$\left[ {\begin{array}{*{20}c} {0.484275 } & {0.5} & { - 0.717968 } & { - 3110.50} \\ { - 0.279596} & {0.866025 } & {0.414519} & {1795.85} \\ {0.829038} & {0 } & {0.559193} & {7112.88} \\ 0 & 0 & 0 & 1 \\ \end{array} } \right]$$	$$\left[ {\begin{array}{*{20}c} {0.118385} & 0 & { - 0.042710} & { - 0.001050} \\ {0.118386} & 0 & { - 0.042711} & { - 0.001052} \\ { - 0.042709} & 0 & {0.118386} & {0.000294} \\ \end{array} } \right]$$
(− 30°, − 36°, 3°, 6739, − 35°, 0)	$$\left[ {\begin{array}{*{20}c} {0.324419 } & {0.5} & { - 0.802965 } & {{ } - 3112.54} \\ { - 0.187303} & {0.866025} & {0.463592} & {1797.03} \\ {0.927183} & 0 & {0.374607} & { 7111.86} \\ 0 & 0 & 0 & 1 \\ \end{array} } \right]$$	$$\left[ {\begin{array}{*{20}c} { - 0.250787} & 0 & {0.070620 } & { - 0.000395} \\ { - 0.250787} & 0 & {0.070619} & { - 0.000397} \\ {0.070620} & 0 & { - 0.250787} & {0.000151} \\ \end{array} } \right]$$
(− 30°, − 61°, 28°, 5953, − 26°, 0)	$$\left[ {\begin{array}{*{20}c} {0.446036 } & {0.5} & { - 0.742329} & {{ } - 3118.21} \\ { - 0.257519} & {0.866025 } & {0.428584} & {1800.30} \\ {0.857167} & {0 } & {0.515038} & {7108.39} \\ 0 & 0 & 0 & 1 \\ \end{array} } \right]$$	$$\left[ {\begin{array}{*{20}c} {0.030075} & 0 & { - 0.010228} & {0.001426} \\ {0.030076} & 0 & { - 0.010229} & {0.001424} \\ { - 0.010228} & 0 & {0.030076} & { - 0.000338} \\ \end{array} } \right]$$
(− 30°, − 49°, 14°, 6233, − 20°, 3°)	$$\left[ {\begin{array}{*{20}c} {0.522219} & {0.473318} & { - 0.709406 } & { - 3120.63} \\ { - 0.241071} & {0.879848} & {0.4095760} & {1801.69} \\ {0.818029} & { - 0.042871 } & {0.573576} & {7104.54} \\ 0 & 0 & 0 & 1 \\ \end{array} } \right]$$	$$\left[ {\begin{array}{*{20}c} {0.206012} & { - 0.053364} & { - 0.054125} & {0.002202} \\ { - 0.035716} & {0.015961 } & { - 0.054125} & {0.002200} \\ { - 0.055420} & 0 & {0.147153} & { - 0.000878} \\ \end{array} } \right]$$

As shown in Table 2, the AGA method achieves an orientation accuracy better than 10^−6^ and a position accuracy better than 10^−2^. Conversely, Table 3 indicates that the GA method achieves an orientation accuracy lower than 10^−2^ and a position accuracy better than 10^−2^. A comprehensive comparison reveals that the overall solution accuracy of AGA is significantly superior to that of GA. This enhancement is attributed to the AGA’s ability to reduce the number of unknown variables or narrow the solution search space through algebraic analysis, thereby substantially improving the algorithm’s computational precision.

### Joint trajectory planning

According to the operational workflow, the installation robot’s motion cycle comprises three primary phases: moving from the initial position to the grasping position, transporting the HC from the grasping position to the installation position, and returning to the grasping position. This study focuses on trajectory planning for the second phase: the transport from the grasping position to the installation position.

Upon completing the grasp of the HC, the robot’s joint configuration q1 is defined as

$${\mathrm{q1}} = \left[ {{7}0^{ \circ } , - {6}0^{ \circ } , - {5}^{ \circ } ,{331}0, - {3}0^{ \circ } ,0} \right]$$

When the HC is positioned near the tunnel ceiling for installation, the joint variables q2, calculated using the AGA method, are listed in Table 2. To optimize energy and efficiency, q2 is selected to minimize the path length from q1.

$${\mathrm{q2}} = [ - {3}0^{ \circ } , - {59}^{ \circ } ,{26}^{ \circ } ,{6}0{14}, - {27}^{ \circ } ,0]$$

**Fig. 9:**
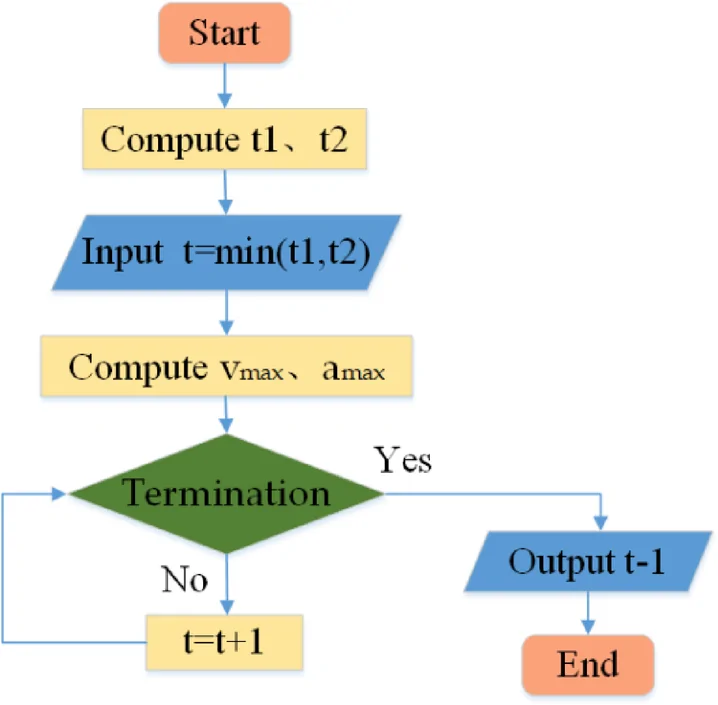
Flowchart of the computation method.

Building on the shortest-path principle, this paper proposes a computational method to obtain a time-optimal polynomial interpolation trajectory, as shown in Fig. 9. This approach minimizes the total motion time ${\mathrm{t}}_{f}$ by maximizing the joint velocity ${\dot{\mathrm{q}}}\left( {\mathrm{t}} \right)$ or acceleration $\ddot{q}\left( {\mathrm{t}} \right)$ within their physical limits.

The values for t1 and t2 are determined by Eq. (9). Since the final motion time ${\mathrm{t}}_{f}$ must satisfy ${\mathrm{t}}_{f}$ > min(t1, t2), we initialize the search variable t with min(t1, t2). The algorithm then incrementally increases t to calculate the corresponding maximum velocity v_max_ and maximum acceleration a_max_. The process continues until either v_max_ reaches the velocity limit ${\dot{\mathrm{q}}}\left( {\mathrm{t}} \right)_{\max }$ or a_max_ reaches the acceleration limit $\ddot{q}\left( {\mathrm{t}} \right)_{\max }$. At this convergence point, the value of t from the previous iteration step is output as the minimum feasible motion time ${\mathrm{t}}_{f}$.

9 $${\mathrm{t}}1 = \frac{{{\dot{\mathrm{q}}}\left( {\mathrm{t}} \right)_{{{\mathrm{max}}}} }}{{ \ddot{q}\left( {\mathrm{t}} \right)_{{{\mathrm{max}}}} }},\;{\mathrm{t}}2 = \frac{q2 - q1}{{{\dot{\mathrm{q}}}\left( {\mathrm{t}} \right)_{{{\mathrm{max}}}} }}$$

As the robot transitions from configuration q1 to q2 with joint J6 remaining stationary, trajectory planning is required only for the remaining five joints. The maximum allowable joint velocities and accelerations are listed in Table 4.

**Table 4:** The maximum values of joint velocity and acceleration.

	joint J1	joint J2	joint J3	joint J4	joint J5	joint J6
${\dot{\mathrm{q}}}\left( {\mathrm{t}} \right)_{{{\mathrm{max}}}}$	1.5°/s	1.5°/s	1.5°/s	50 mm/s	2.0°/s	2.0°/s
$\ddot{q}\left( {\mathrm{t}} \right)_{{{\mathrm{max}}}}$	0.05°/s^2^	0.10°/s^2^	0.10°/s^2^	2 mm/s^2^	0.20°/s^2^	0.20°/s^2^

#### Fifth-order polynomial interpolation

The joint trajectories are generated using a fifth-order polynomial interpolation method. The time-dependent profiles of joint position, velocity, acceleration, and jerk are defined by Eq. (10).

10 $$\left\{ {\begin{array}{*{20}l} {q\left( {\mathrm{t}} \right) = {\mathrm{a}}_{0} + {\mathrm{a}}_{1} t + {\mathrm{a}}_{2} {\mathrm{t}}^{2} + {\mathrm{a}}_{3} {\mathrm{t}}^{3} + {\mathrm{a}}_{4} {\mathrm{t}}^{4} + {\mathrm{a}}_{5} {\mathrm{t}}^{5} } \hfill \\ {{\dot{\mathrm{q}}}\left( {\mathrm{t}} \right) = {\mathrm{a}}_{1} + 2{\mathrm{a}}_{2} t + 3{\mathrm{a}}_{3} {\mathrm{t}}^{2} + 4{\mathrm{a}}_{4} {\mathrm{t}}^{3} + 5{\mathrm{a}}_{5} {\mathrm{t}}^{4} } \hfill \\ {{\ddot{\mathrm{q}}}\left( {\mathrm{t}} \right) = 2{\mathrm{a}}_{2} + 6{\mathrm{a}}_{3} t + 12{\mathrm{a}}_{4} {\mathrm{t}}^{2} + 20{\mathrm{a}}_{5} {\mathrm{t}}^{3} } \hfill \\ {{\dddot{\mathrm{q}}}\left( {\mathrm{t}} \right) = 6{\mathrm{a}}_{3} + 24{\mathrm{a}}_{4} t + 60{\mathrm{a}}_{5} {\mathrm{t}}^{2} } \hfill \\ \end{array} } \right.$$

To determine the six unknown coefficients, Eq. (10) must satisfy the boundary constraints specified in Eq. (11), which dictate the position, velocity, and acceleration at both the initial(t = 0) and final (t = ${\mathrm{t}}_{f}$) states.

11 $${\mathrm{q}}\left( 0 \right) = {\mathrm{q}}1{ },\;{\mathrm{q}}\left( {{\mathrm{t}}_{f} } \right) = {\mathrm{q}}2,\;{\dot{\mathrm{q}}}\left( 0 \right) = {\dot{\mathrm{q}}}\left( {{\mathrm{t}}_{f} } \right) = {\ddot{\mathrm{q}}}\left( 0 \right) = {\ddot{\mathrm{q}}}\left( {{\mathrm{t}}_{f} } \right)$$

**Fig. 10:**
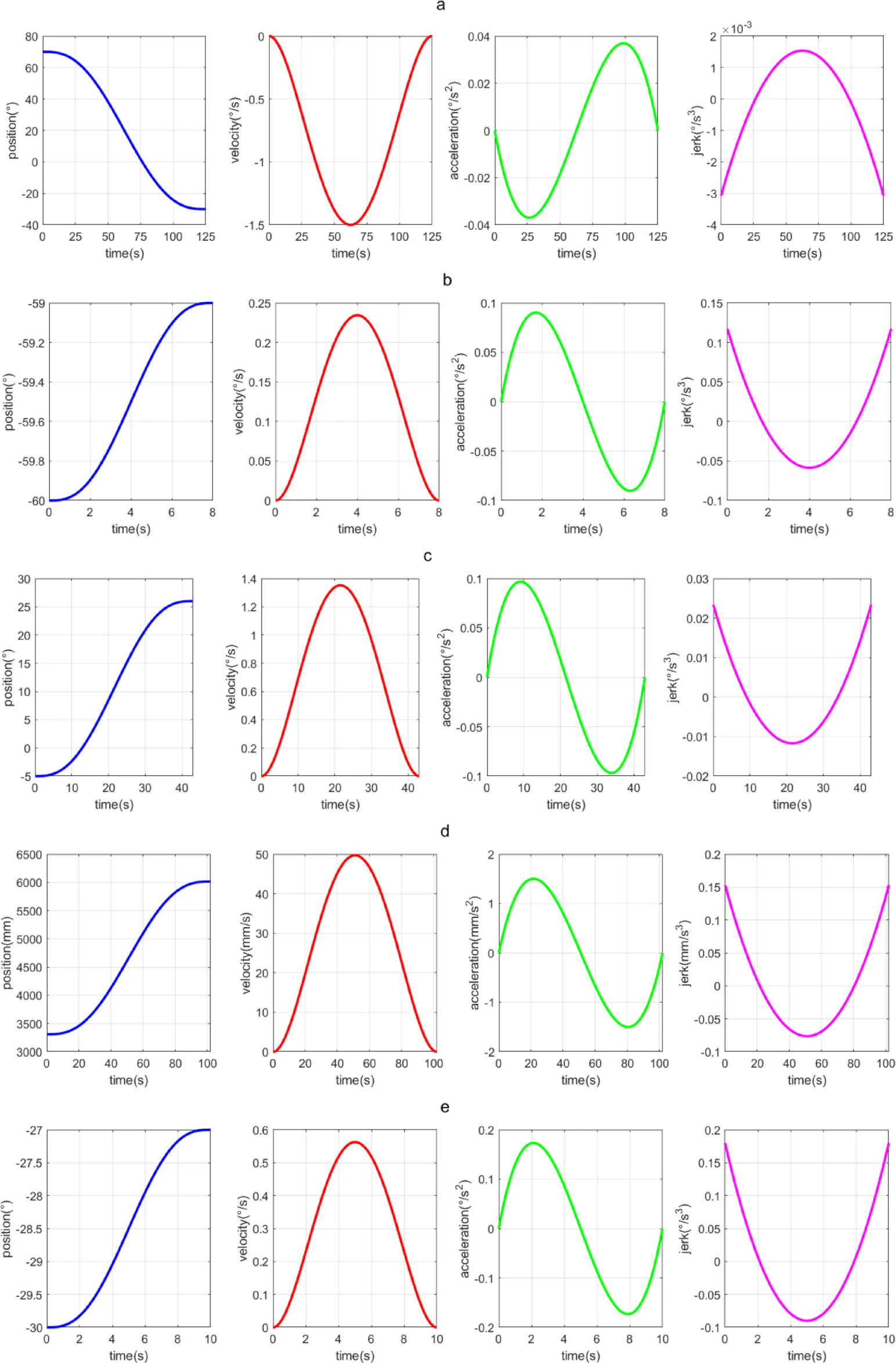
The fifth-order polynomial method. (**a**) Joint J1; (**b**) Joint J2; (**c**) Joint J3; (**d**) Joint J4; (**e**) Joint J5.

Utilizing the proposed computational method, time-optimal fifth-order polynomial trajectories were calculated and simulated. The results are summarized in Table 5 and illustrated in Fig. 10.

**Table 5:** The running results of the fifth-order polynomial interpolation.

	Joint J1	Joint J2	Joint J3	Joint J4	Joint J5
${\mathrm{t}}_{f}$	125	8	43	102	10
v_max_	− 1.5000	0.2344	1.3517	49.7058	0.5625
a_max_	0.0370	0.0902	0.0968	1.5005	0.1732
j_max_	− 0.0031	0.1172	0.0234	0.1529	0.1800

Figure 10 clearly demonstrates that the velocity, acceleration, and jerk profiles are continuous and smooth. While the initial and final velocities and accelerations are zero, the non-zero initial and final jerk values may induce significant impact and vibration, potentially compromising the equipment’s lifespan and precision. As evidenced by Table 5, the motion is limited by different constraints for different joints. Joints 1 and 4 reach their minimum motion times of 125 s and 102 s, respectively, when constrained by maximum velocity. In contrast, Joints 2, 3, and 5 achieve their shortest times of 8 s, 43 s, and 10 s, respectively, when constrained by maximum acceleration.

#### Seventh-order polynomial interpolation

To mitigate vibration and impact, joint trajectories are planned using a seventh-order polynomial interpolation method. The time-dependent profiles of joint position, velocity, acceleration, and jerk are defined by Eq. (12).

12 $$\left\{ {\begin{array}{*{20}l} {q\left( {\mathrm{t}} \right) = {\mathrm{a}}_{0} + {\mathrm{a}}_{1} t + {\mathrm{a}}_{2} {\mathrm{t}}^{2} + {\mathrm{a}}_{3} {\mathrm{t}}^{3} + {\text{ a}}_{4} {\mathrm{t}}^{4} + {\mathrm{a}}_{5} {\mathrm{t}}^{5} + {\mathrm{a}}_{6} {\mathrm{t}}^{6} + {\mathrm{a}}_{7} {\mathrm{t}}^{7} } \hfill \\ {{\dot{\mathrm{q}}}\left( {\mathrm{t}} \right) = {\mathrm{a}}_{1} + 2{\mathrm{a}}_{2} t + 3{\mathrm{a}}_{3} {\mathrm{t}}^{2} + 4{\mathrm{a}}_{4} {\mathrm{t}}^{3} + 5{\mathrm{a}}_{5} {\mathrm{t}}^{4} + 6{\mathrm{a}}_{6} {\mathrm{t}}^{5} + 7{\mathrm{a}}_{7} {\mathrm{t}}^{6} } \hfill \\ {{\ddot{\mathrm{q}}}\left( {\mathrm{t}} \right) = 2{\mathrm{a}}_{2} + 6{\mathrm{a}}_{3} t + 12{\mathrm{a}}_{4} {\mathrm{t}}^{2} + 20{\mathrm{a}}_{5} {\mathrm{t}}^{3} + 30{\mathrm{a}}_{6} {\mathrm{t}}^{4} + 42{\mathrm{a}}_{7} {\mathrm{t}}^{5} } \hfill \\ {{\dddot{\mathrm{q}}}\left( {\mathrm{t}} \right) = 6{\mathrm{a}}_{3} + 24{\mathrm{a}}_{4} t + 60{\mathrm{a}}_{5} {\mathrm{t}}^{2} + 120{\mathrm{a}}_{6} {\mathrm{t}}^{3} + 210{\mathrm{a}}_{7} {\mathrm{t}}^{4} } \hfill \\ \end{array} } \right.$$

To solve for the eight unknown parameters, the Eq. (12) must satisfy the following constraints as shown in Eq. (13), which specify the joint position, velocity, acceleration, jerk at the start position.

To determine the eight unknown coefficients, Eq. (12) must satisfy the boundary constraints specified in Eq. (13). These constraints dictate the values of position, velocity, acceleration, and jerk at both the initial (t = 0) and final (t = ${\mathrm{t}}_{f}$) states.

13 $${\mathrm{q}}\left( 0 \right) = {\mathrm{q}}1{ },\;{\mathrm{q}}\left( {{\mathrm{t}}_{f} } \right) = {\mathrm{q}}2,\;{\dot{\mathrm{q}}}\left( 0 \right) = {\dot{\mathrm{q}}}\left( {{\mathrm{t}}_{f} } \right) = {\ddot{\mathrm{q}}}\left( 0 \right) = {\ddot{\mathrm{q}}}\left( {{\mathrm{t}}_{f} } \right) = \dddot q\left( 0 \right) = \dddot q\left( {{\mathrm{t}}_{f} } \right) = 0$$

**Fig. 11:**
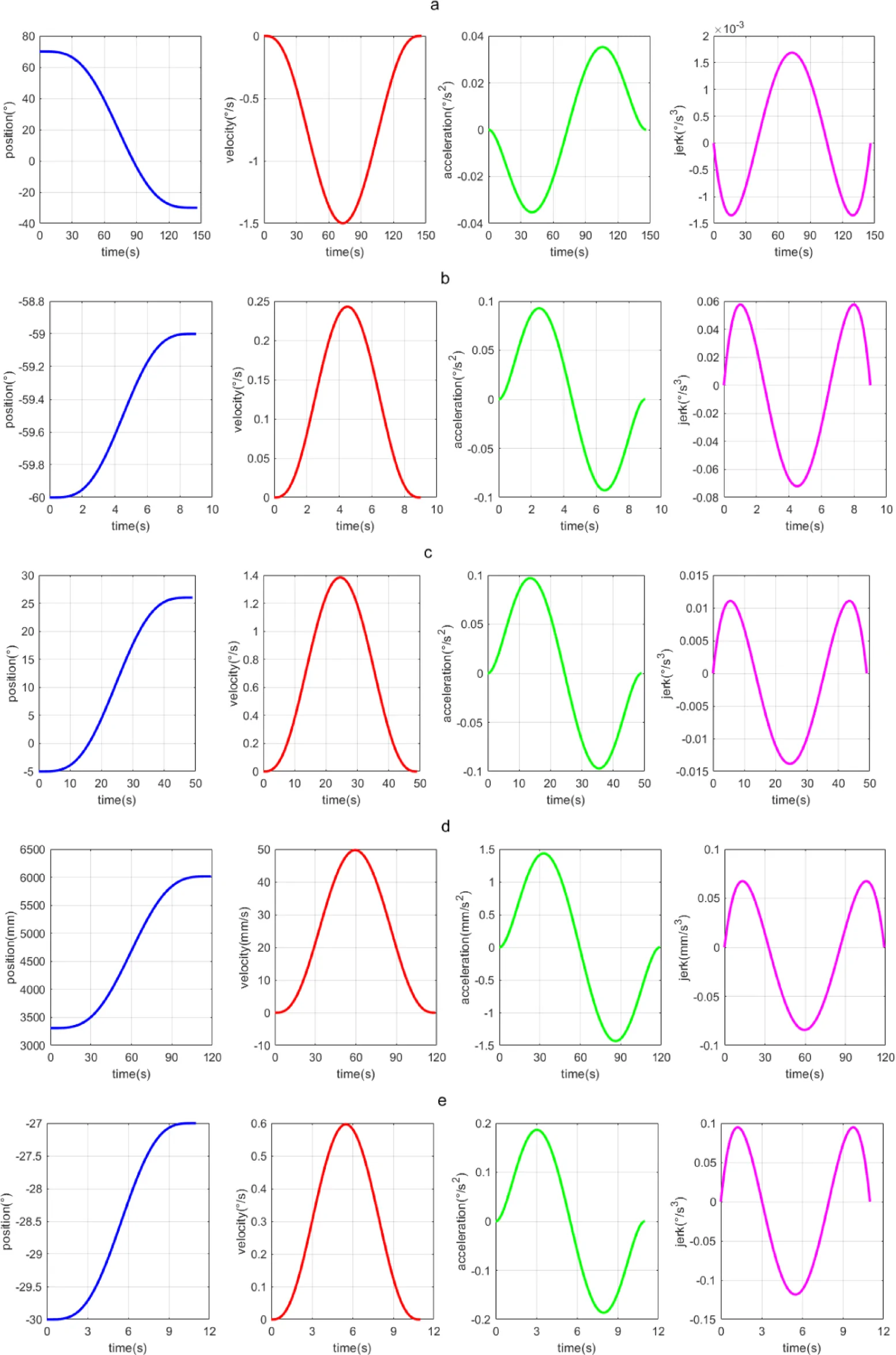
The seventh-order polynomial method. (**a**) Joint J1; (**b**) Joint J2; (**c**) Joint J3; (**d**) Joint J4; (**e**) Joint J5.

Utilizing the proposed computational framework, time-optimal seventh-order polynomial trajectories were calculated and simulated. The resulting data are summarized in Table 6 and illustrated in Fig. 11.

**Table 6:** The running results of the seventh-order polynomial method.

	joint J1	joint J2	joint J3	joint J4	joint J5
${\mathrm{t}}_{f}$	146	9	49	119	11
v_max_	− 1.4982	0.2431	1.3839	49.7059 mm/s	0.5966
a_max_	0.0352	0.0928	0.0970	1.4346 mm/s^2^	0.1863
j_max_	0.0017	− 0.0720	− 0.0138	− 0.0842	− 0.1183

Figure 11 demonstrates that the trajectory profiles are smooth and continuous without discontinuities. The joint velocity, acceleration, and jerk all start and end at zero, which effectively mitigates mechanical shock and wear, reduces noise and vibration, improves motion stability, and extends lifespan.

As evidenced by Table 6, the minimum motion times for Joints 1 and 4 are 146 s and 119 s, respectively, under velocity-limited conditions. For Joints 2, 3, and 5, the shortest motion times are 9 s, 49 s, and 11 s, respectively, under acceleration-limited conditions.

Based on the comprehensive results above, the maximum velocity and acceleration capabilities of both methods are comparable. However, while the fifth-order polynomial interpolation yields a shorter motion duration, the seventh-order method produces a significantly lower maximum jerk with no abrupt changes. Consequently, the seventh-order polynomial interpolation better satisfies the vibration suppression requirements for the installation robot.

## Experiment and discussion

To ensure quality and safety, critical components must undergo basic testing after production. Upon passing tests, they are assembled together for overall testing to verify operational capability.

### The end-effector and robotic arm testing

**Fig. 12:**
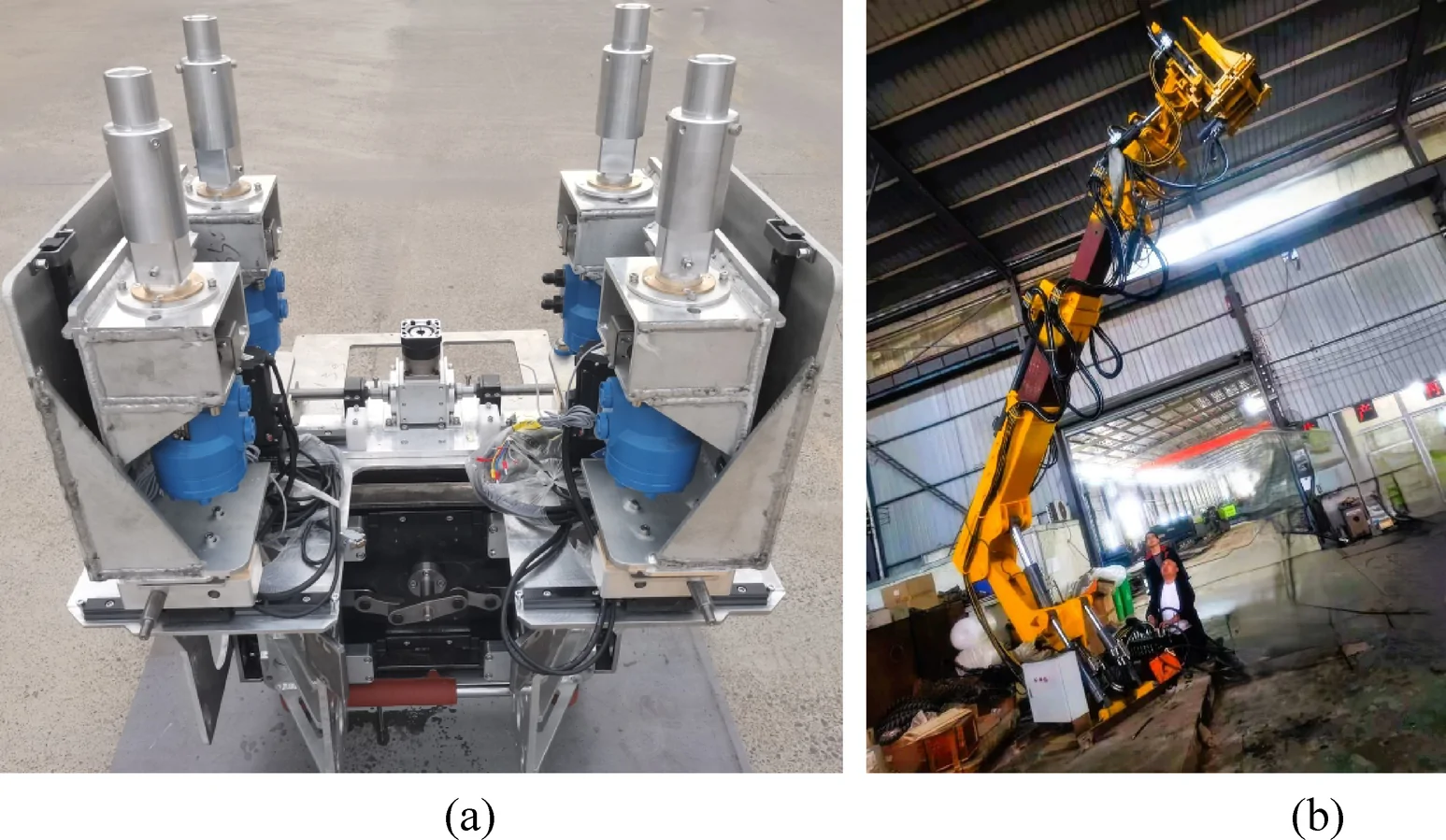
The physical picture of the end-effector and robotic arm. (**a**) The end-effector; (**b**) The robotic arm.

All components were procured and processed to assemble the end-effector, as shown in Fig. 12a. According to design specifications, the total weight was required not to exceed 250 kg. The main mechanisms were weighed, and the results, presented in Table S2, indicate a total weight of 222.5 kg. Subsequently, motion tests were conducted with the hydraulic station powering the end-effector’s operation (Fig. S4). The results demonstrate that the end-effector can successfully perform gripping and clamping actions on HC.

Following the design documents, the robotic arm was assembled as illustrated in Fig. 12b. Joint working ranges were tested in the workshop, confirming that the robotic arm meets all operational requirements.

Finally, the end-effector and robotic arm were integrated to form the complete installation robot. The process of grasping HC and transporting it to the installation position was tested, as shown in Fig. S5. The results confirm that the robot can successfully grasp HC and transport it to the designated installation position.

### Installation robot testing

#### Test environment setup

**Fig. 13:**
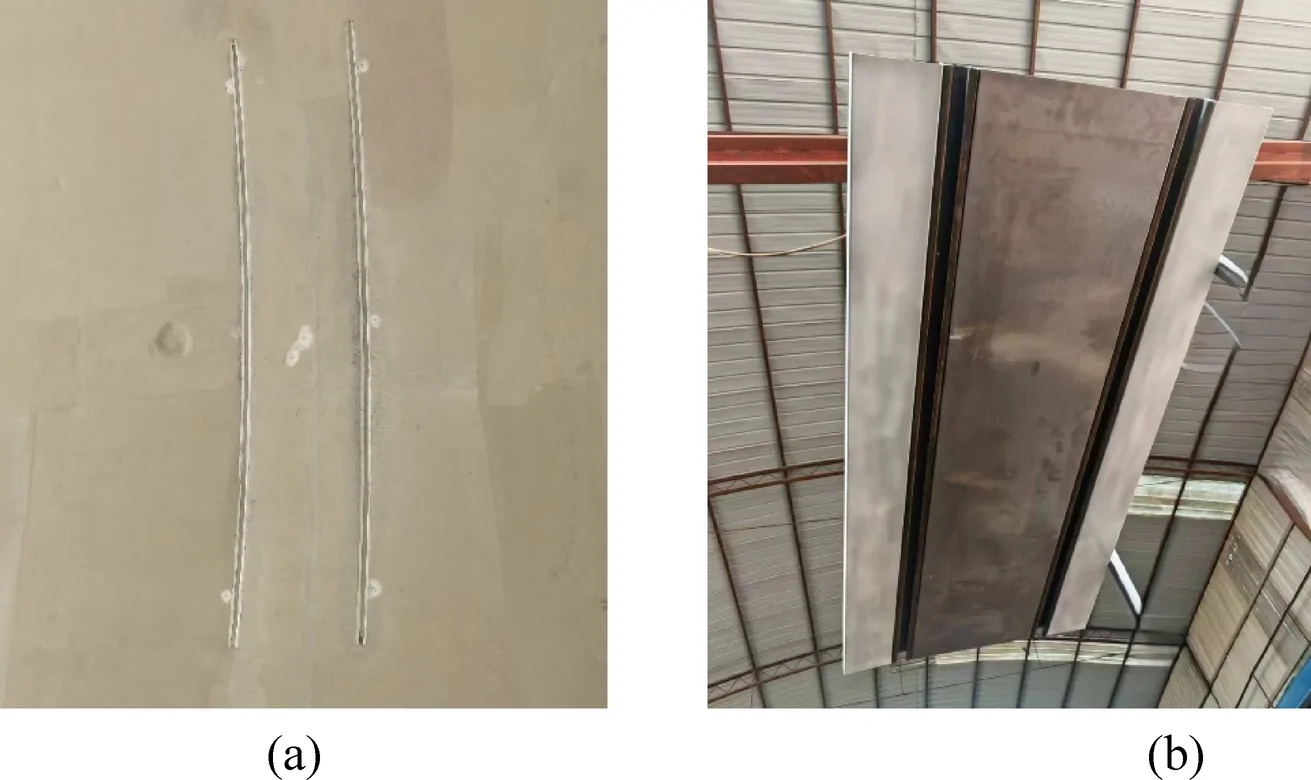
The installation grooves. (**a**) Real photograph; (**b**) Simulated model.

The overall dimensions of the equipment are approximately 15 m in length, 2.55 m in width, and 4 m in height, resulting in a substantial footprint. Additionally, given that the robot’s operating height exceeds 8 m, a dedicated indoor facility was selected for the experiments. The equipment was transported to this site to establish the experimental environment. In the experiment, two parallel channels were machined from metal plates to simulate the installation grooves at the tunnel crown. These metal plates were fixed at a height of 8 m to approximate the tunnel environment, as illustrated in Fig. 13.

#### Operational efficiency test

**Fig. 14:**
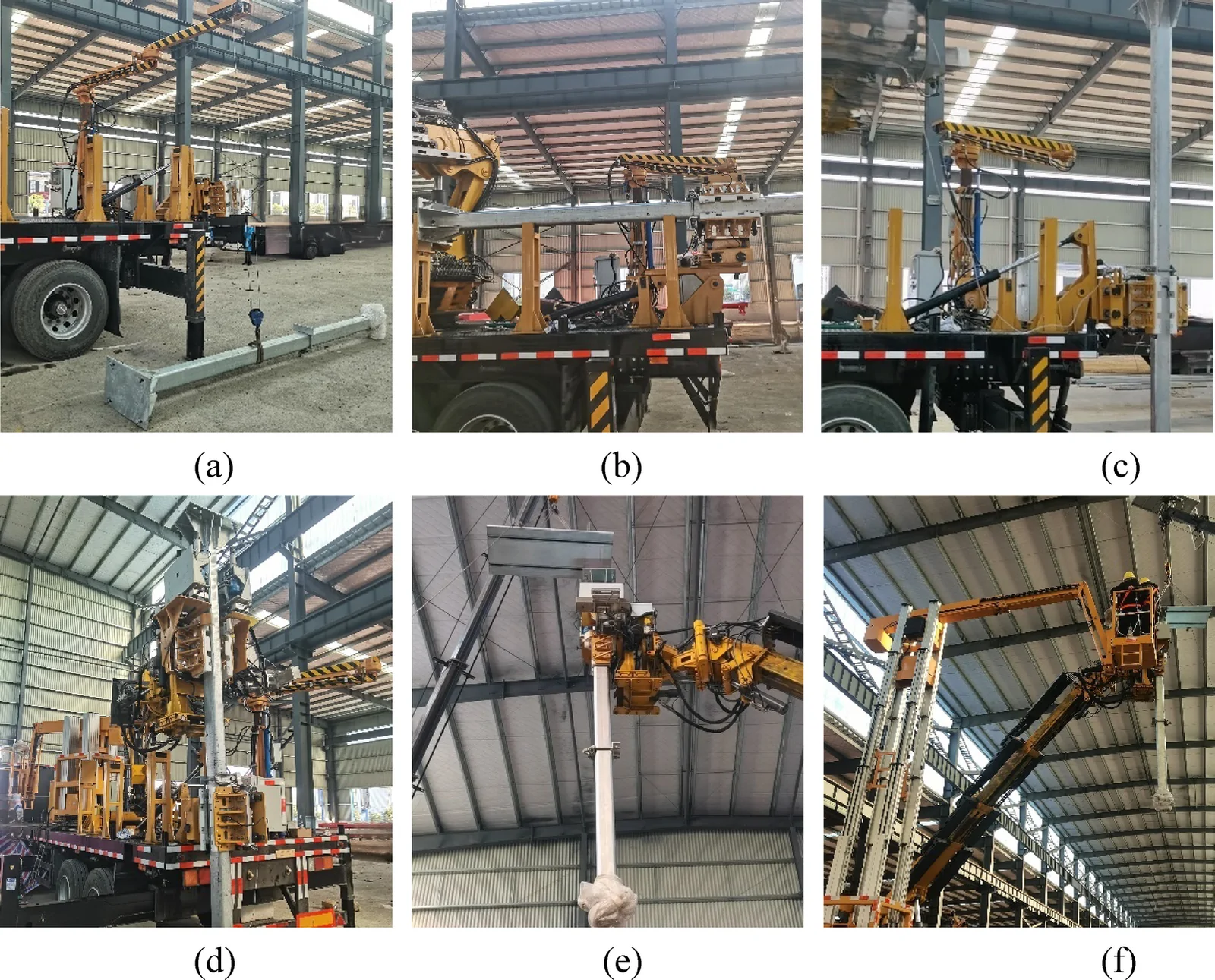
The operation procedure. (**a**) Parking; (**b**) Hoisting and storage; (**c**) Rotating; (**d**) Griping; (**e**) Lifting; (**f**) Installing.

The operational efficiency of the automated and mechanized HC installation scheme utilizing the installation robot was evaluated. The workflow is illustrated in the Fig. 14. Based on multiple trials, the approximate time consumption for each step is as follows: parking and preparation take (180 s), the cantilever crane hoists the HC from the ground to the storage rack(120 s), the turning mechanism grasps and rotates the HC 90° (50 s), the installation robot lifts the HC to the installation position (620 s), installation and inspection(240 s), the gripper releases the load and the robot retracts to its initial position(120 s).The entire process takes approximately 22 min and 50 s. Compared to the roughly 1 h required for manual installation, representing an efficiency improvement of approximately 1.7 times.

#### Stability and accuracy test

**Fig. 15:**
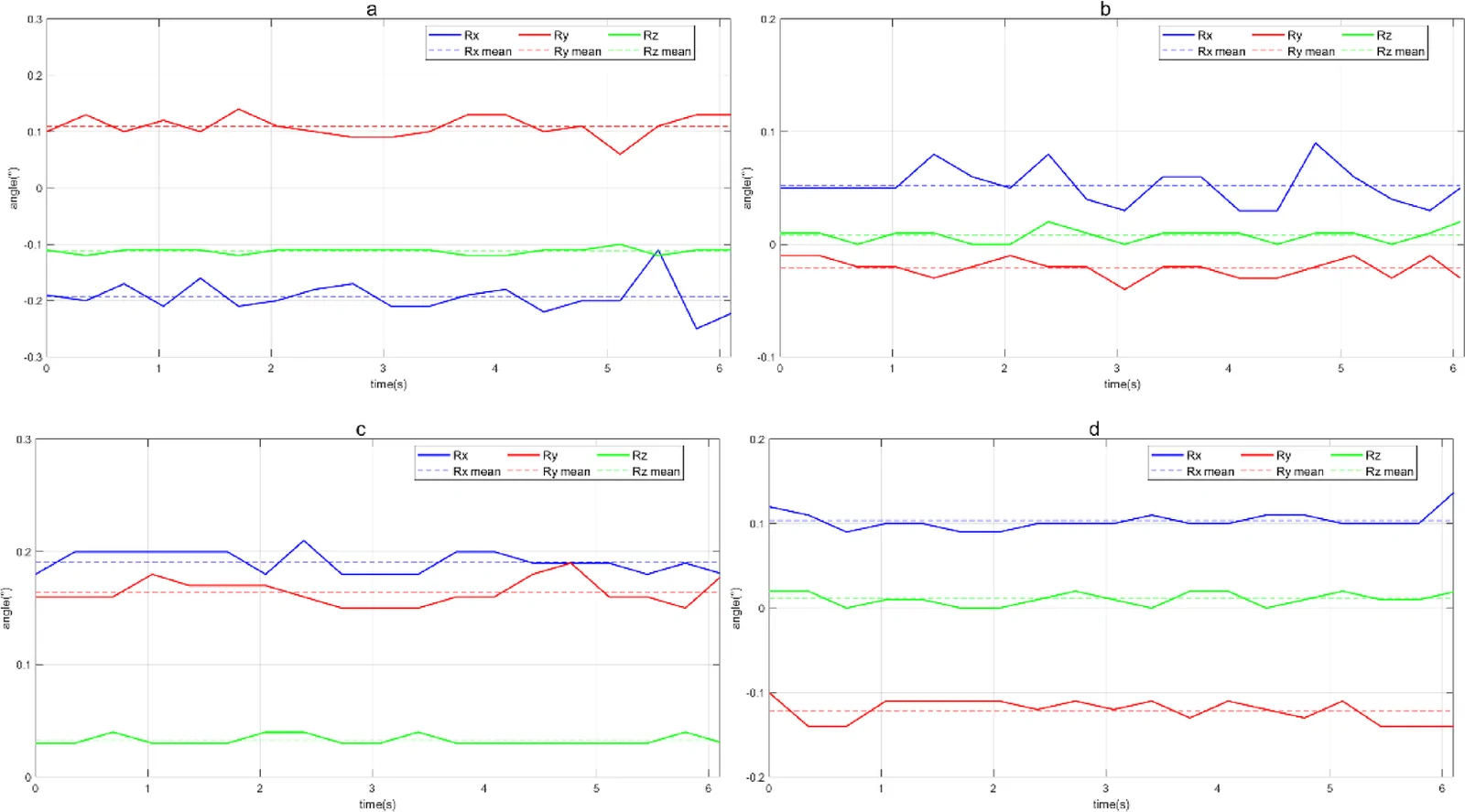
Variation curves of Rx, Ry, and Rz recorded in four tests. (**a**) G1, (**b**) G2, (**c**) G3, (**d**) G4.

Experiments were conducted to evaluate the stability and positioning accuracy of the installation robot. A Cartesian coordinate system was established with the target installation position as the origin, where the x-axis aligns with the installation groove, the y-axis perpendicular to the x-axis within the horizontal plane, and the z-axis represents the vertical direction. To ensure successful HC installation, the length, width, and height of its base plate must align with the x-axis, y-axis and z-axis, respectively. Consequently, the angular deviations of the HC orientation from these axes, denoted as Rx, Ry, and Rz, serve as key metrics for assessing the end-effector’s accuracy at the installation point. During the experiments, the values of the deviation angles Rx, Ry, and Rz were measured after the installation robot lifted the HC to the target position and the equipment stabilized for a period of time. Multiple independent tests were conducted on-site, and the results of four selected trials are presented in Fig. 15.

**Fig. 16:**
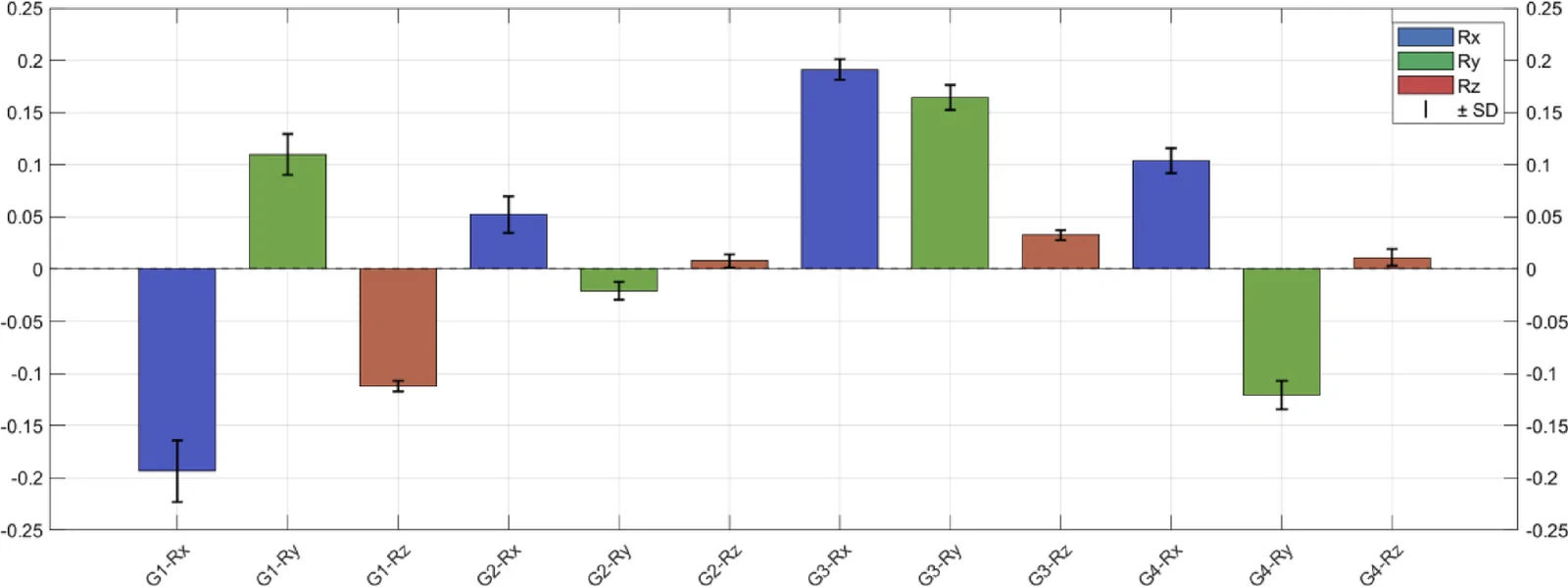
Comparison of means and standard deviations of Rx, Ry, and Rz in four tests.

To comprehensively evaluate the distribution characteristics of the test data, statistical analysis was performed on the Rx, Ry, and Rz data obtained from four independent tests. The minimum, maximum, range, mean, median, and standard deviation (SD) were calculated, as presented in Table 7. Furthermore, a Mean-SD distribution plot (Mean ± SD), as shown in Fig. 16, was generated to provide a more intuitive comparison of the central tendency and dispersion, where the bar charts represent the means and the error bars indicate ± 1 SD.

**Table 7:** The Statistical values of Rx, Ry, Rz.

		Minimum	Maximum	Range	Mean	Median	SD
G1	Rx	− 0.25	− 0.11	0.14	− 0.1937	− 0.2000	0.0293
	Ry	0.06	0.14	0.08	0.1095	0.1100	0.0196
	Rz	− 0.12	0.10	0.02	− 0.1121	− 0.1100	0.0054
G2	Rx	0.03	0.09	0.06	0.0521	0.0500	0.0175
	Ry	− 0.04	− 0.01	0.03	− 0.0211	− 0.0200	0.0088
	Rz	0.00	0.02	0.02	0.0079	0.0100	0.0063
G3	Rx	0.18	0.21	0.03	0.1911	0.1900	0.0099
	Ry	0.15	0.19	0.04	0.1642	0.1600	0.0117
	Rz	0.03	0.04	0.01	0.0326	0.0300	0.0045
G4	Rx	0.09	0.14	0.05	0.1037	0.1000	0.0116
	Ry	− 0.14	− 0.10	0.04	− 0.1211	− 0.1200	0.0137
	Rz	0.00	0.02	0.02	0.0111	0.0100	0.0081

As shown in Table 6 and Fig. 16, for G1, Rx exhibits a high dispersion and poor stability (Range = 0.14, SD = 0.0293), whereas Rz demonstrates the best stability (Range = 0.02, SD = 0.0054). Similarly, Rx shows relatively high instability (Range = 0.06, SD = 0.0175), while Rz maintains the highest stability (Range = 0.02, SD = 0.0063) in G2. For G3, Ry displays poorer stability due to higher dispersion (Range = 0.04, SD = 0.0117), compared to Rz, which exhibits the best stability (Range = 0.01, SD = 0.0045). Finally, G4 follows a similar trend where Ry indicates the least stability (Range = 0.04, SD = 0.0137) and Rz remains the most stable (Range = 0.02, SD = 0.0081). Collectively, G3 exhibits the smallest ranges (≤ 0.04) and SD (≤ 0.0117) across all three components, Rx, Ry, and Rz, demonstrating the best stability among the four groups. Notably, Rz shows consistently smaller fluctuations across all groups, with both range (≤ 0.02) and SD (≤ 0.0081) remaining at low levels, indicating significantly better stability than Rx and Ry.

Although the ideal values for Rx, Ry, and Rz are defined as zero, the engineering specifications define the tolerance threshold as $\left| {R_{x} } \right| \le 0.15, \left| {R_{y} } \right| \le 0.15, \left| {R_{z} } \right| \le 0.15.$ To assess the accuracy of robot, this paper systematically examines the means in conjunction with their corresponding 95% confidence intervals (CI) relative to the designated tolerance range of [− 0.15,0.15], as summarized in Table 8.

**Table 8:** Accuracy analysis results based on tolerance range of [− 0.15, 0.15].

		Mean	Mean within tolerance?	95% CI	95% CI within tolerance?
G1	Rx	− 0.1937	No	[− 0.2078, − 0.1796]	No
	Ry	0.1095	Yes	[0.1000, 0.1189]	Yes
	Rz	− 0.1121	Yes	[− 0.1147, − 0.1095]	Yes
G2	Rx	0.0521	Yes	[0.0437, 0.0605]	Yes
	Ry	− 0.0211	Yes	[− 0.0254, − 0.0168]	Yes
	Rz	0.0079	Yes	[0.0049, 0.0109]	Yes
G3	Rx	0.1911	No	[0.1863, 0.1958]	No
	Ry	0.1642	No	[0.1586, 0.1698]	No
	Rz	0.0326	Yes	[0.0305, 0.0348]	Yes
G4	Rx	0.1037	Yes	[0.0981, 0.1093]	Yes
	Ry	− 0.1211	Yes	[− 0.1277, − 0.1144]	Yes
	Rz	0.0111	Yes	[0.0072, 0.0150]	Yes

As shown in Table 8, for G1, the means and their corresponding 95% CI for Ry and Rz are situated within tolerance range, while Rx fails to meet the criterion. For G3, only Rz’s mean and 95% CI fall within tolerance range, with both Rx and Ry failing to comply. In contrast, for G2 and G4, the means and 95% CI across all three axes are entirely contained within tolerance range. Notably, a more detailed comparison reveals that both mean and the 95% CI of G2 are clustered more closely around zero, indicating optimal accuracy. Additionally, the means and 95% CI of Rz across all groups fall completely within tolerance range, demonstrating that its accuracy is significantly superior to that of Rx and Ry.

In summary, the results of the four tests indicate that, under the ideal engineering criteria of $\left| {R_{x} } \right| \le 0.15,\left| {R_{y} } \right| \le 0.15,\left| {R_{z} } \right| \le 0.15$, G2 exhibits optimal accuracy and favorable stability, thereby demonstrating the best comprehensive performance. Furthermore, the Rz demonstrated significantly superior accuracy and stability compared to Rx and Ry, suggesting that the system’s control and error suppression capabilities are more mature in the vertical direction or the corresponding attitude component. Conversely, the Rx and Ry directions remain the critical constraints limiting further enhancement of overall system performance. These experimental results demonstrate that the current robotic system possesses the engineering capability to meet the requirements of HC installation tasks. In particular, the results from G2 validate that a favorable combination of high accuracy and robust stability can be achieved. Furthermore, these findings establish a definitive direction for subsequent optimization efforts. Specifically, targeted improvements in parameter tuning, error compensation, and stability enhancement for the Rx and Ry axes are essential to enhance the robot’s accuracy consistency and operational reliability under different operating conditions.

## Conclusion

Based on the design, theoretical analysis, algorithm optimization and experimental verification of the integrated operational equipment, this paper draws the following core conclusions:

The designed integrated operational equipment can effectively achieve grabbing, lifting and installation. It successfully addresses the challenges of high physical labor intensity, significant safety risks, and low operational efficiency inherent in traditional methods, providing reliable mechanical support for installing HC in tunnels.

The forward kinematics model, established using the MD–H parameter method, proves to be accurate and reliable. Simulation results demonstrate that the robot’s workspace fully satisfies operational range requirements, laying a solid theoretical foundation for its practical application.

The proposed inverse kinematics solution, which combines algebraic and genetic algorithm, significantly enhances solution accuracy. Experimental verification indicates a rotation matrix error of. Experimental verification shows that its rotation matrix error is less than 10 × 10^−6^ and the position coordinate error is less than 10 × 10^−3^. The accuracy meets the engineering requirements, effectively overcoming the accuracy limitations of traditional inverse kinematics methods.

The time-optimal trajectory planning algorithm, based on seventh-order polynomial interpolation, effectively mitigates impact and vibration. It ensures smooth joint motion while simultaneously maximizing operational efficiency.

The experimental results conclusively demonstrate that the proposed robotic operation scheme and the designed integrated equipment can successfully execute the installation of catenary hanging columns in tunnels, thereby validating the feasibility and practicality of the overall research framework.

## Supplementary Information

Below is the link to the electronic supplementary material.

Supplementary Material 1

## Data Availability

The data supporting the results of this study are presented in the main text. Raw data and codes are available from the corresponding author upon reasonable request.
