# Supplementary material for: Design, analysis, and experimental research on installation robot for hanging column
Source: Sci Rep. 2026 Jun 9;16:26223. doi: 10.1038/s41598-026-54684-w (PMC13493813; doi:10.1038/s41598-026-54684-w)
Supplement: Supplementary file 1 [file 41598_2026_54684_MOESM1_ESM.pdf]

Supplementary material

Design, Analysis, and Experimental Research on Installation Robot for Hanging Column

Xiaoqiang Wang[1,2], Xijing Zhu[1,*], Xiang Li [2], and Jingyu Yang[2]

1 School of Mechanical Engineering, North University of China, Taiyuan 030051, China;
2 713th Research Institute of China State Shipbuilding Corporation Limited, Zhengzhou 450015, China;

Table S1. The end pose matrix

Joint variables	By equation (3)	By model in MATLAB
(29°，−30°，25°，4620，-40°，70°)	$\begin{bmatrix} -0.2440 & -0.7470 & -0.6184 & 53.74 \\ 0.9391 & -0.0230 & 0.3428 & 29.79 \\ 0.2418 & -0.6645 & 0.7071 & 6365 \\ 0 & 0 & 0 & 1 \end{bmatrix}$	$\begin{bmatrix} -0.2440 & -0.7470 & -0.6184 & 53.74 \\ 0.9391 & -0.0230 & 0.3428 & 29.79 \\ 0.2418 & -0.6645 & 0.7071 & 6365 \\ 0 & 0 & 0 & 1 \end{bmatrix}$
(-15°，-50°，13°，5810，-25°，-23°)	$\begin{bmatrix} 0.3163 & 0.4154 & -0.8529 & -3508 \\ -0.4893 & 0.8417 & 0.2285 & 940 \\ 0.8128 & 0.3450 & 0.4695 & 6518 \\ 0 & 0 & 0 & 1 \end{bmatrix}$	$\begin{bmatrix} 0.3163 & 0.4154 & -0.8529 & -3508 \\ -0.4893 & 0.8417 & 0.2285 & 940 \\ 0.8128 & 0.3450 & 0.4695 & 6518 \\ 0 & 0 & 0 & 1 \end{bmatrix}$
(65°，-65°，-5°，6793，-10°，14°)	$\begin{bmatrix} -0.1480 & -0.8971 & -0.4162 & -2964 \\ 0.2549 & 0.3720 & -0.8925 & -6357 \\ 0.9556 & -0.2382 & 0.1736 & 4033 \\ 0 & 0 & 0 & 1 \end{bmatrix}$	$\begin{bmatrix} -0.1480 & -0.8971 & -0.4162 & -2964 \\ 0.2549 & 0.3720 & -0.8925 & -6357 \\ 0.9556 & -0.2382 & 0.1736 & 4033 \\ 0 & 0 & 0 & 1 \end{bmatrix}$

Table S2. Weight of the end-effector

	name	weight(kg)
1	hinge clamping mechanism	89.7
2	alignment adjustment	50.3
3	tightening assembly	67.5
4	other accessories	15

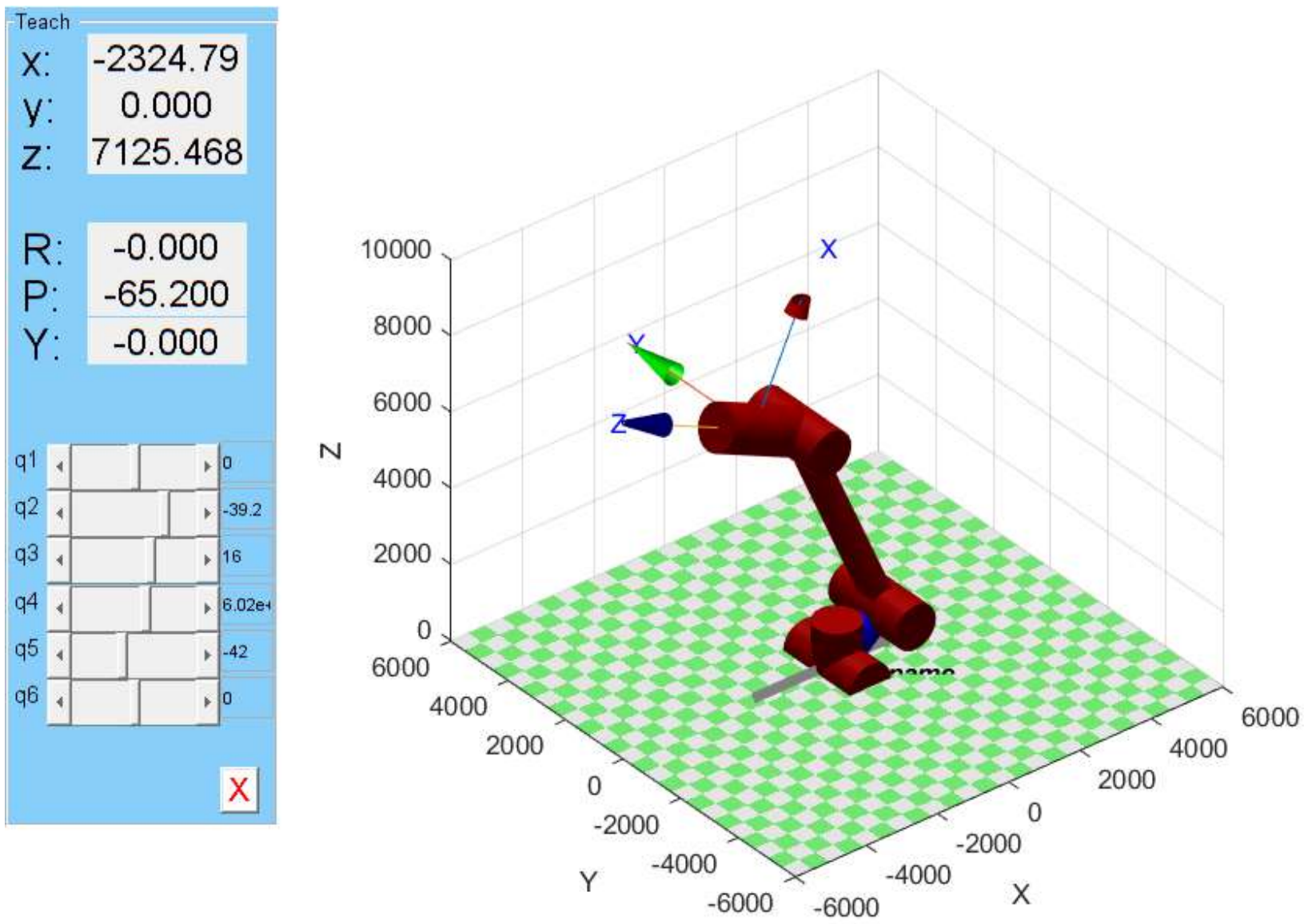

Figure S1. Simulation model of installation robot.

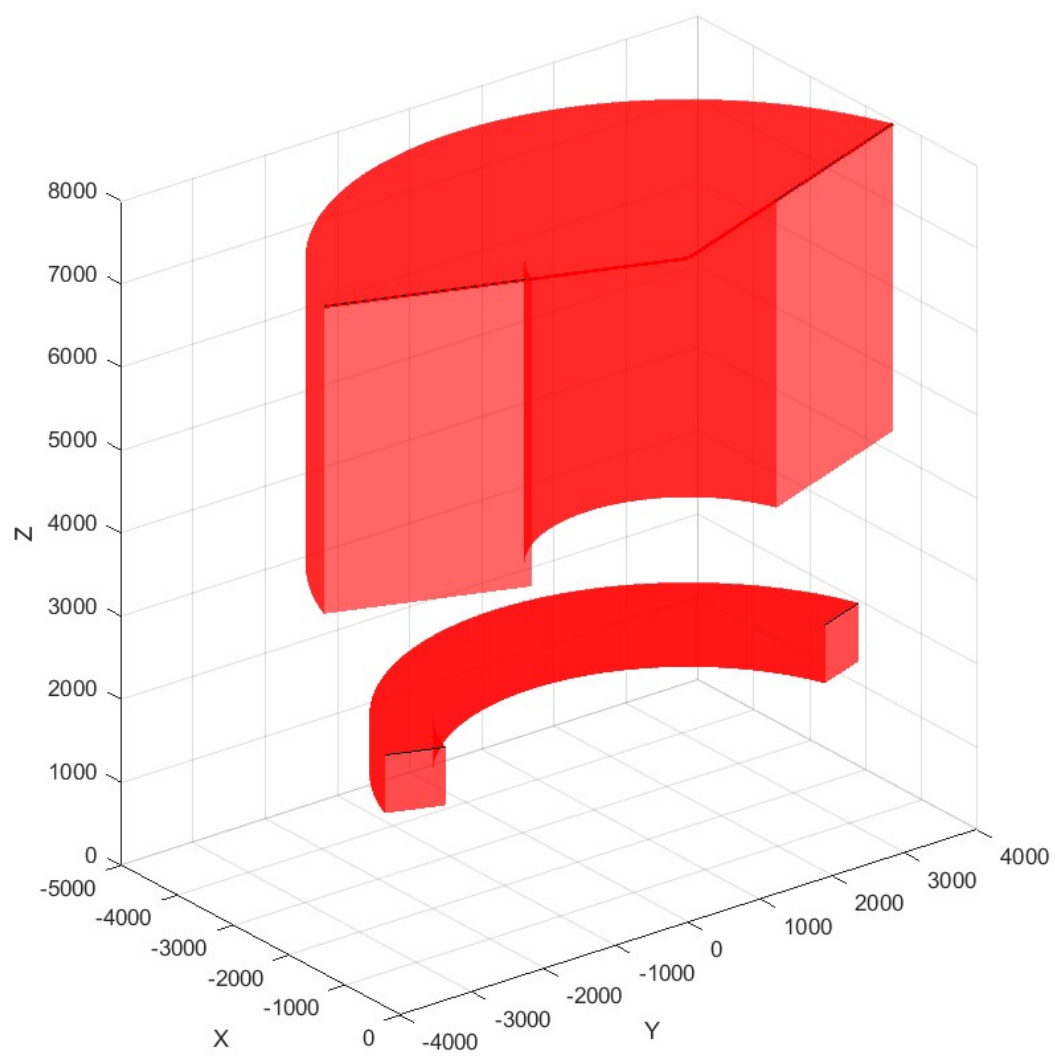

Figure S2. Working range requirement of installation robot

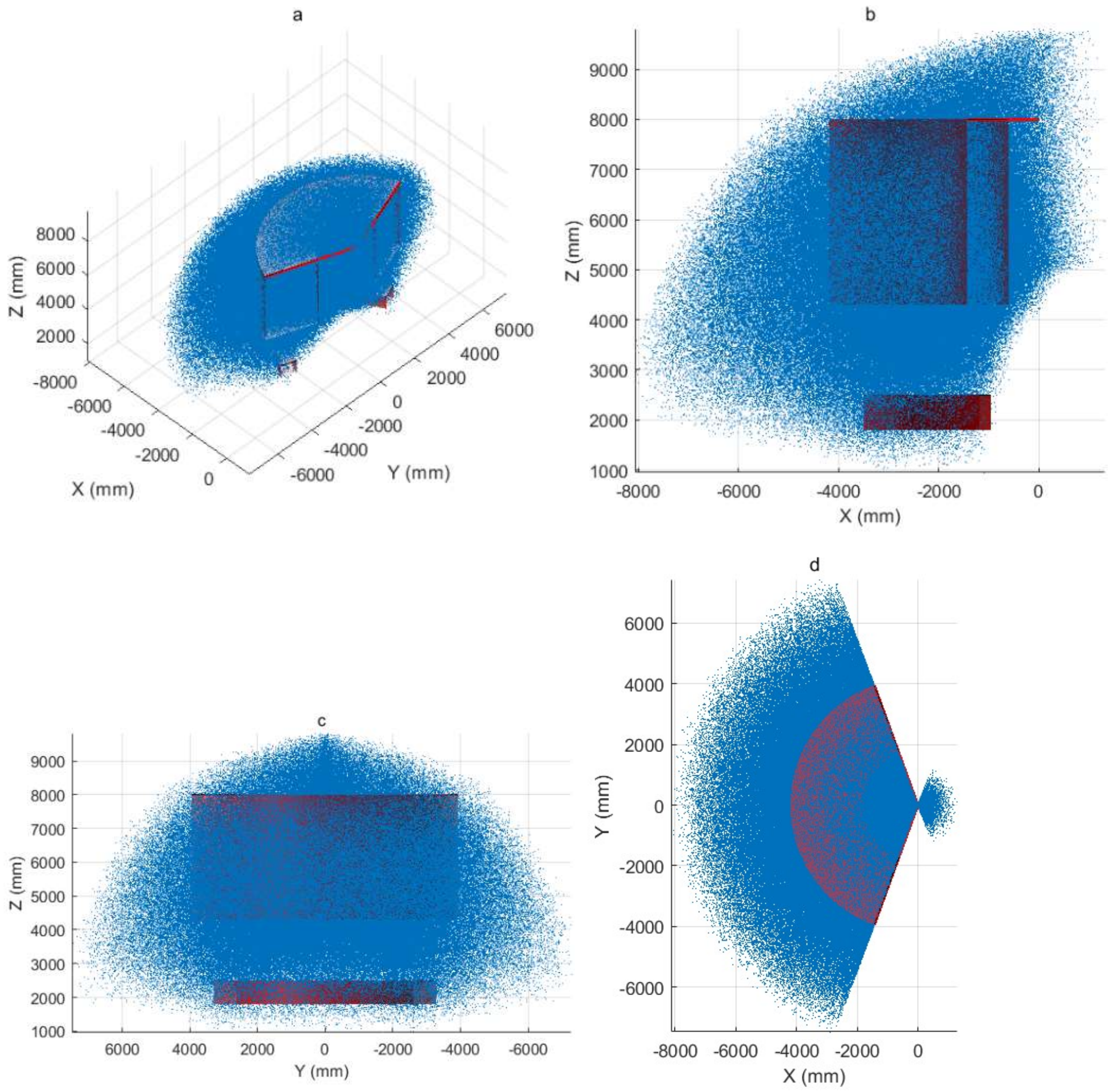

Figure S3. Workspace of installation robot. (a) Global Workspace; (b) X0Z Plane; (c) YOZ Plane; (d) X0Y Plane.

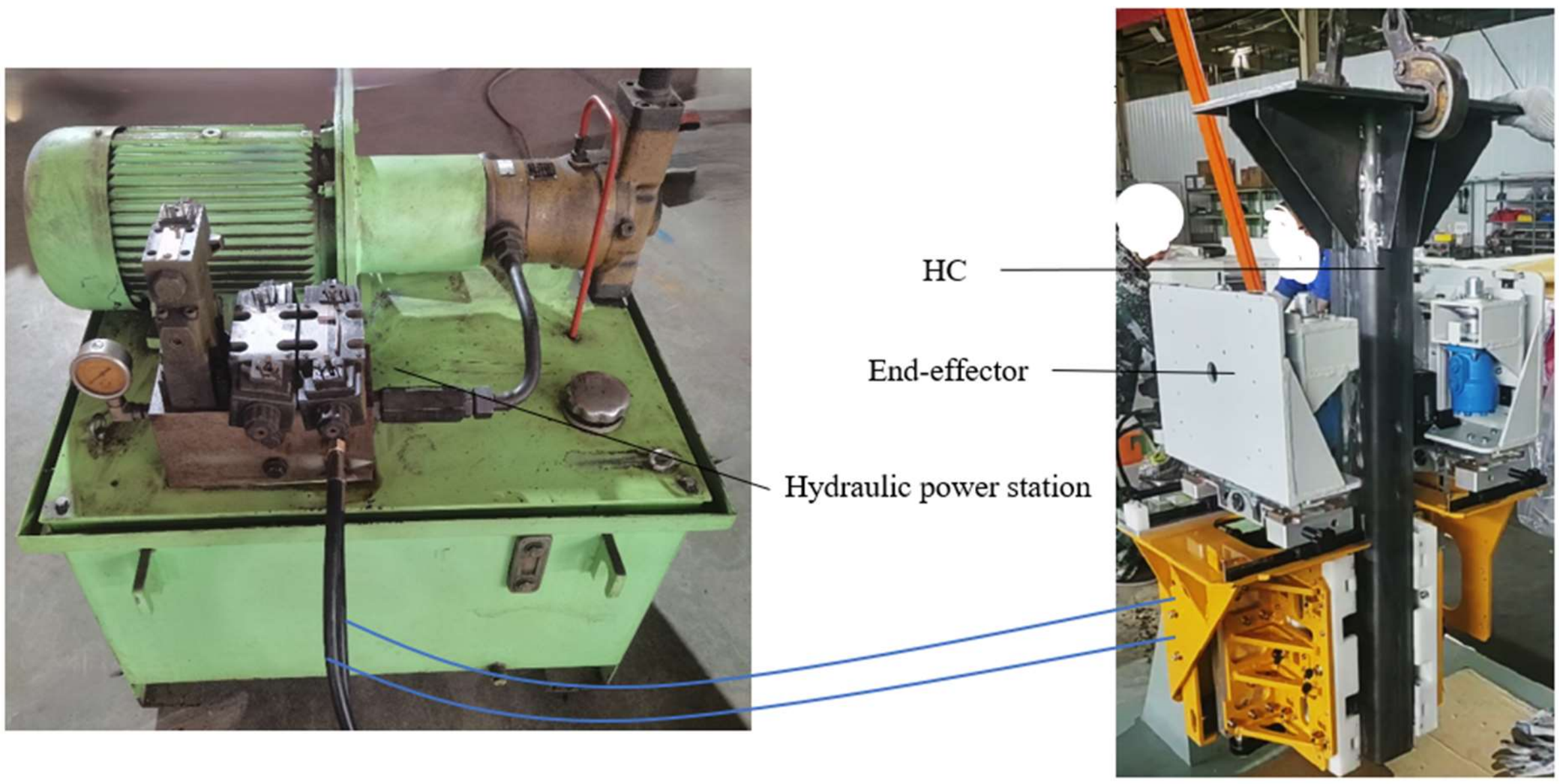

Figure S4. Picture of the end-effector testing.

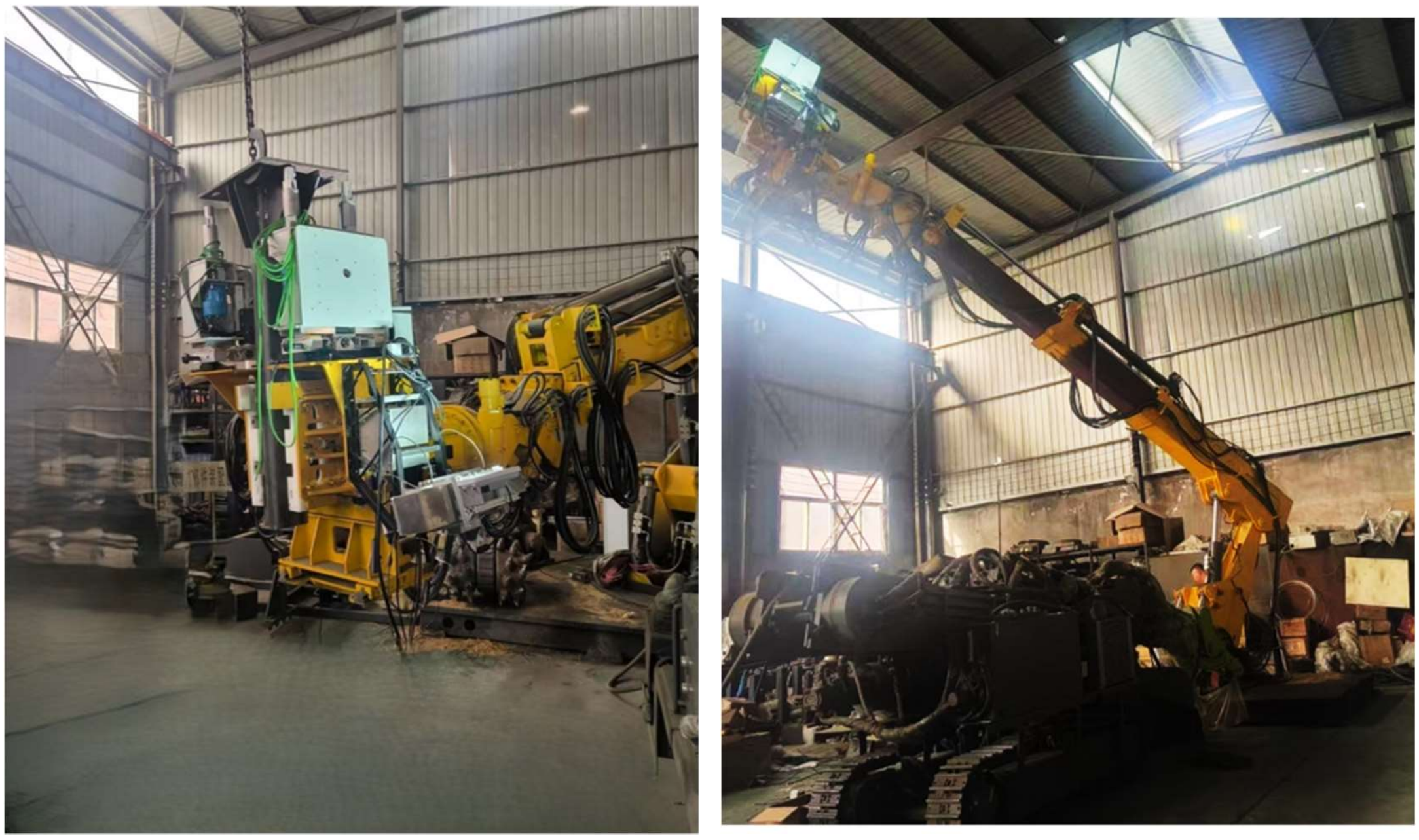

(a) (b)

Figure S5. Picture of installation robot testing. (a) Grabbing HC; (b) Transporting HC to installation position
